# Characterising the Gut Microbiomes in Wild and Captive Short-Beaked Echidnas Reveals Diet-Associated Changes

**DOI:** 10.3389/fmicb.2022.687115

**Published:** 2022-06-30

**Authors:** Tahlia Perry, Ella West, Raphael Eisenhofer, Alan Stenhouse, Isabella Wilson, Belinda Laming, Peggy Rismiller, Michelle Shaw, Frank Grützner

**Affiliations:** ^1^The Environment Institute, School of Biological Sciences, The University of Adelaide, Adelaide, SA, Australia; ^2^Centre of Excellence for Australian Biodiversity and Heritage, The University of Adelaide, Adelaide, SA, Australia; ^3^Perth Zoo, South Perth, WA, Australia; ^4^Pelican Lagoon Research and Wildlife Centre, Penneshaw, SA, Australia; ^5^Taronga Wildlife Nutrition Centre, Taronga Conservation Society Australia, Mosman, NSW, Australia

**Keywords:** EchidnaCSI, Australia, captive, herbivore, insects, nutrition, digestive physiology

## Abstract

The gut microbiome plays a vital role in health and wellbeing of animals, and an increasing number of studies are investigating microbiome changes in wild and managed populations to improve conservation and welfare. The short-beaked echidna (*Tachyglossus aculeatus*) is an iconic Australian species, the most widespread native mammal, and commonly held in zoos. Echidnas are cryptic animals, and much is still unknown about many aspects of their biology. Furthermore, some wild echidna populations are under threat, while echidnas held in captivity can have severe gastric health problems. Here, we used citizen science and zoos to collect echidna scats from across Australia to perform the largest gut microbiome study on any native Australian animal. Using 16S rRNA gene metabarcoding of scat samples, we characterised and compared the gut microbiomes of echidnas in wild (*n* = 159) and managed (*n* = 44) populations, which were fed four different diets. Wild echidna samples were highly variable, yet commonly dominated by soil and plant-fermenting bacteria, while echidnas in captivity were dominated by gut commensals and plant-fermenting bacteria, suggesting plant matter may play a significant role in echidna diet. This work demonstrates significant differences between zoo held and wild echidnas, as well as managed animals on different diets, revealing that diet is important in shaping the gut microbiomes in echidnas. This first analysis of echidna gut microbiome highlights extensive microbial diversity in wild echidnas and changes in microbiome composition in managed populations. This is a first step towards using microbiome analysis to better understand diet, gastrointestinal biology, and improve management in these iconic animals.

## Introduction

The influence of the gut microbiome on host health has been well established in humans, with many diseases and health problems associated with microbial dysbiosis, including obesity, diabetes, and bowel disease (Turnbaugh et al., 2006; Frank et al., 2007; Wen et al., 2008; Cho and Blaser, 2012). How microbiomes affect the health in non-human animals has only recently been investigated, but is recognised as vital for wildlife conservation and captive management (Bahrndorff et al., 2016; McKenzie et al., 2017; Trevelline et al., 2019; Chong et al., 2020). Australia is home to an exceptional suite of endemic and evolutionarily diverse mammals, from all three major clades (eutherians, marsupials, and monotremes). To date, microbiome studies have been performed on a subset of native Australian mammals, most of which have been marsupials from geographically isolated populations (Brice et al., 2019; Chong et al., 2020; Eisenhofer et al., 2021). To date, the monotreme (egg-laying mammal) microbiome has not been investigated.

The short-beaked echidna (*Tachyglossus aculeatus*) is the most widespread native mammal in Australia, found across all types of habitats from desert and temperate regions, to snowy alpine (Archer, 1983; Rismiller and Grutzner, 2019) and one of three egg-laying mammals alongside the platypus and long-beaked echidna. Although echidnas are an iconic Australian species, we have relatively little information about most wild populations due to their cryptic and solitary lifestyles and large home-ranges (Abensperg-Traun, 1991; Rismiller and Mckelvey, 1994). The only long-term studies of echidnas are on Kangaroo Island, South Australia, and specific areas in Tasmania (Rismiller, 1992; Nicol and Andersen, 2002, 2007; Rismiller and McKelvey, 2003). Therefore, using genetic techniques to investigate their gut microbiome across a wide range of habitats will provide information about the biology of these remarkable egg-laying mammals and may be a good indicator of health. This is becoming more urgent for echidnas as work on Kangaroo Island for more than 25 years has recorded the impacts of feral animals and environmental changes, which led to the subspecies (*Tachyglossus aculeatus multiaculeatus*) being recognised as endangered (EPBC Act, 2015 ‘Conservation Advice *T. aculeatus multiaculeatus* Kangaroo Island echidna’). Echidnas face the same threats across all of Australia, raising concerns about their conservation status Australia-wide. A better understanding of wild echidna populations is an important step towards long-term conservation.

Echidnas are commonly held in zoos across the world but frequently suffer from gastrointestinal problems and poor captive breeding success, which are believed to be related to diet in captivity. In managed populations, diets for echidnas were initially based on carnivore (i.e. cat and dog) models, as this was believed to be comparatively the most similar digestive system and therefore, have similar nutrient requirements (Augee et al., 2006; Stannard et al., 2017). However, echidnas on these diets frequently display nutrition-related problems such as diarrhoea, gastritis, cystitis, and obesity (Stannard et al., 2017). Therefore, new diets have been developed to address these problems. Key changes in diet include an increase in protein and fat to better reflect the natural insectivorous diet and higher fibre content to account for the high soil and organic matter echidnas usually ingest when foraging (Griffiths and Greenslade, 1990; Stannard et al., 2017), with recent diets balancing macro- and micronutrients to meet expected requirements. There are four diets currently being used and monitored in Australia; however, how these different diets affect the gut microbiome of echidnas is yet to be investigated.

Diet is a major determinant of the bacterial communities in the gut microbiome, with many phylogenetically distant mammals clustering together as carnivores, omnivores, and herbivores, with herbivores even forming distinct groups of foregut and hindgut fermenters based on the location and the composition of these microbes living in their gut (Ley et al., 2008). Echidnas are often mistakenly characterised as myrmecophagous animals (exclusively ant and termite eaters) but instead eat a wide variety of invertebrates including (but not limited to) ants, termites, beetles, worms, and a range of insect larvae (Griffiths, 1968; Smith et al., 1989; Sprent and Nicol, 2016) and have even been associated with the distribution of mycorrhizal fungi (Feuerherdt et al., 2005). Echidnas are opportunistic foragers, and their diets will change depending on the food availability, season and temperature (Smith et al., 1989; Sprent and Nicol, 2016); however, echidnas diet across their wide habitat types has yet to be characterised in detail. In some parts of Australia and times of the year when echidnas’ diet consists of mostly ants and termites, their gut microbiomes may be similar to myrmecophagous species (Delsuc et al., 2014); however, gut microbial communities are likely to be based on the echidnas’ geographical location and the availability of food.

Faeces (or scats) are commonly used for studying animals’ gut microbiomes as they can be noninvasively collected and do not require the animal itself to be present in order to collect the sample. In zoos, scat samples can be easily collected by animal care staff; however, collecting an adequate number of samples across multiple locations for wild populations can be difficult. Some studies have successfully collected scat or other material (such as swabs or ticks) to analyse microbiomes from large and geographically dispersed datasets through citizen science initiatives (Hulcr et al., 2012; Klimenko et al., 2018; McDonald et al., 2018; Chauhan et al., 2020). As echidnas cover a vast geographic range, citizen scientists have been enlisted to collect echidna scats through the project EchidnaCSI (Echidna Conservation Science Initiative; Stenhouse et al., 2021; Perry et al., 2022), leading to the largest echidna material collection to date. Echidna scats can be easily identified as they are a smooth cylindrical shape, approximately 2 cm in diameter and mostly consist of soil and undigested exoskeletons of prey items (Augee et al., 2006; Rismiller and Grutzner, 2019).

Here, we present the first comparative gut microbiome study for wild and managed populations of echidnas, which is the largest and most geographically spread microbiome study of any Australian mammal to date. We aimed to (1) characterise the gut microbiome of wild echidnas across their diverse habitats in Australia; (2) investigate how captivity influences the echidna gut microbiome; and (3) determine if different diets alter the gut microbiome in zoo-held echidnas.

## Materials and Methods

### Wild Echidnas Faecal Sample Collection and Metadata

**Figure 1:**
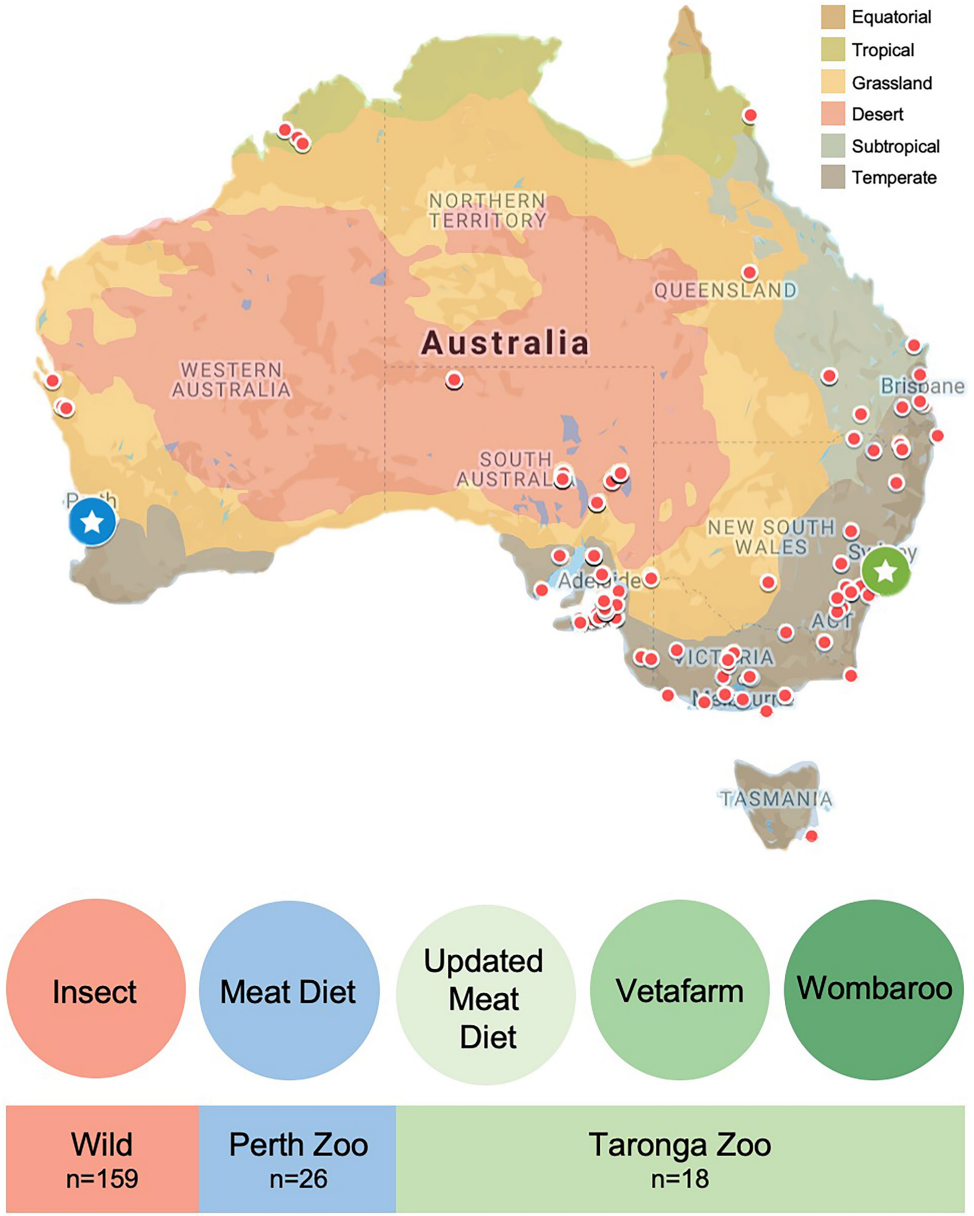
Location and diet information for faecal samples collected from wild and captive echidnas in this study. Red dots on map indicate locations of faecal samples collected from wild echidnas, diet labelled as ‘insect’ for simplicity; blue circle with star is the location of Perth Zoo, where faecal samples were collected from echidnas that were fed exclusively the Meat diet; green circle with star is the location of Taronga Zoo, where faecal samples were collected from echidnas fed three different diets: Updated Meat Diet (UMD), Vetafarm diet and Wombaroo diet. Map is coloured according to Australian climate zones.

Faecal samples from wild echidnas were collected through a collaborative effort with volunteers throughout Australia as a part of the citizen science project: Echidna Conservation Science Initiative (EchidnaCSI; Perry et al., 2022). Participants were instructed to download the EchidnaCSI app, which housed photographs and detailed instructions on how to identify an echidna scat. Participants were then instructed to take a photograph of the echidna scat through the EchidnaCSI app when the sample was found so that the date, time, and GPS location could be matched to the physical samples. Once a photograph was taken, the app directed the participant to place the faecal sample in a clean zip-lock bag without touching the faecal samples, or instead using gloves, to avoid contamination. Samples were shipped immediately to The University of Adelaide and then stored in the freezer. A total of 159 wild samples from across Australia were used in this study from a large variety of locations and environments, and across all major climate regions (Figure 1; Supplementary Table S1). Scats were confirmed as belonging to echidna either by visual inspection as they have a distinct appearance, or by PCR of an ‘echidna specific’ dloop region of the mitochondria as outlined in (Perry et al., 2022). Most scats were collected opportunistically and were in the environment for an unknown time prior to collection, which may alter the taxonomy of the faecal microbiome. To assess the differences between collection times and microbiome composition in echidna scats, we worked closely with a subset of citizen scientists in South Australia, who collected fresh scats (within a day of depositing faeces) from their properties where echidnas often frequented (sample names with * in Supplementary Table S1). As samples were collected opportunistically, diet information for wild samples was unknown, for simplicity we have labelled the wild samples as having an ‘insect’ diet (Figure 1). Based on GPS coordinates, each sample was given metadata associated with its location (e.g. climate, land use, anthropogenic biomes, land cover; Supplementary Table S1) by using the Atlas of Living Australia’s Spatial Portal.^1^

### Captive Echidnas Faecal Sample Collection

Faecal samples were collected from managed echidnas in two locations: Perth Zoo and Taronga Zoo (Figure 1; Supplementary Table S2). Faecal material was collected from nine echidnas at Perth Zoo, Western Australia (31.9755°S, 115.8523°E), where biological triplicate faecal samples were collected from each individual (collected consecutively across 3 days, except for echidna identified as ‘Nyingarn’ where only two samples were collected; *n* = 26). Faecal material was collected from 10 echidnas from Taronga Zoo, New South Wales (33.8435°S, 151.2413°E; sample number varied per individual; *n* = 18). Samples were collected by zoo personnel, where fresh faecal samples (within 1 day of echidna depositing faeces) were handled with gloves and placed in a clean plastic zip-lock bag or screw-capped tube and then, immediately frozen. Samples were shipped to The University of Adelaide (from Perth Zoo on dry ice and from Taronga Zoo on ice) and again stored immediately in the freezer.

Echidnas at Perth Zoo were only fed the Meat diet, which consisted of lean beef mince, microcrystalline cellulose, hardboiled egg, banana, multivitamin supplement with iron (Pentavite), calcium carbonate, mealworms, and water. Echidnas at Taronga Zoo were fed three different diets (see Supplementary Table S3 for comprehensive diet information): The Updated Meat Diet (UMD), which is similar to the Meat diet fed in Perth Zoo; Vetafarm diet, manufactured by Vetafarm (VETAFARM AUSTRALIA PTY LTD, Wagga Wagga, NSW), where the main sources of protein are meat meal, corn, and soy; and Wombaroo diet, manufactured by Wombaroo Food Products (PITTENWEEN PTY LTD, Mount Barker, SA), which contains meat meal, soy, and whey protein isolate as the protein sources. The Meat and Updated Meat Diets were both made in-house, while the Vetafarm and Wombaroo diets were made commercially.

### DNA Extraction

Total genomic DNA was extracted from 203 unique faecal samples using the Qiagen QIAamp Mini Stool Kit (Qiagen, Hilden, Germany) as per the manufacturer’s protocol, apart from some details outlined below. A further two faecal samples underwent triplicate DNA extractions to test the reproducibility of faecal microbiome using one-third of the sample for each extraction (samples CO232181, CO232182, CO232183, CO25218, CO252181, CO252182). The extractions took place in a Flow Cabinet Biological Safe Level 2 that was cleaned with 10% bleach (sodium hypochlorite) to reduce contamination. A third of the scat sample was crushed up in the presence of liquid nitrogen using a mortar and pestle, using only the core of the scat to avoid environmental contamination, prior to adding the sample to InhibiteX buffer. Next, samples were centrifuged at 20,000 *g* for 3 min and ~1 ml eluate transferred to a new 1.5 ml tube. Samples were again centrifuged at 20,000 *g* for 1 min and ~700 μl of eluate transferred to a new 1.5 ml tube, carefully avoiding any transfer of physical material. Samples were centrifuged one last time at 20,000 *g* for 1 min and 600 μl added to 25 μl Proteinase K such as in the protocol; from here, the rest of the manufacturer’s protocol was followed.

### PCR Amplification

All samples were PCR amplified and uniquely barcoded, using primers targeting the V4 region of the bacteria 16S ribosomal RNA (rRNA) gene (Caporaso et al., 2011). DNA was amplified with the primer pair 515F (5′-AATGATACGGCGACCAC CGAGATCTACACTATGGTAATTGTGTGCCAGCMGCCGCG GTAA-3′) and uniquely barcoded 806R (5′-CAAGCAGAAGA CGGCATACGAGATnnnnnnnnnnnnAGTCAGTCAGCCGGAC TACHVG GGT WTCTAAT-3′; Integrated DNA Technologies). Single reactions of 18.7 μl dH2O, 2.5 μl 10× HiFi buffer, 1 μl 50 mM MgSO_4_, 0.1 μl Platinum Taq DNA Polymerase (ThermoFisher), 0.2 μl 100 mM dNTP, mix, 0.5 μl of 10 μM forward primer, 1 μl of 5 μM reverse primer and 1 μl DNA. DNA was amplified using an initial denaturation at 94°C for 3 min, followed by 35 cycles of denaturation at 94°C for 45 s, annealing at 50°C for 1 min, elongation at 68°C for 90 s, with final adenylation for 10 min at 68°C, in line with the Earth Microbiome Protocol (Thompson et al., 2017).

Gel electrophoresis was carried out for each PCR reaction on a 2.5% agarose gel to ensure the samples contained library constructs of the desired length (~390 bp). Each sample was then quantified using Qubit 2.0 Fluorometer and pooled to equimolar concentration. Pooled samples were cleaned following the Agencourt AMPure XP PCR purification protocol (Beckman Coulter), quantified and quality checked by the LabChip® GX Touch™ nucleic acid analyser. The wild and captive samples were sequenced separately; all wild samples and captive samples were pooled separately to a final concentration of 4 nM and were run on the Illumina Miseq (wild samples: v2, 2 × 250 bp, 15 million read pair output; captive samples: Nano, 2 × 250 bp, 1 million read pair output) at ACRF (Australian Cancer Research Foundation) Cancer Genomics Facility.

### Data Processing and Statistical Analyses

DNA sequencing data were processed and analysed using QIIME2 v2022.2 (Bolyen et al., 2019). Demultiplexed paired-end sequence reads were merged, quality filtered and denoised into amplicon sequence variants (ASVs) using the *deblur* plugin (Amir et al., 2017) and trim length of 252 bp. For wild samples, the feature table was rarefied to 5,800, and for captive samples the feature table was rarefied to a depth of 1,300, using the minimum number of sequences per sample for diversity analysis. Wild and captive samples were also combined to compare between the two groups; when this occurred, the samples were rarefied to a depth of 2,000 to retain most captive samples and have a valid sequencing depth for appropriate analyses (see Supplementary Figure S1 for rarefaction curves). Representative sequences were assigned taxonomy using the *feature-classifier* plugin (Naïve Bayesian approach) on the pre-trained SILVA (Quast et al., 2013) 138 V4 region classifier (Bokulich et al., 2018), phylogenetic trees were built using FastTree. Alpha diversity was assessed by diversity metrics including observed ASVs, Faith’s phylogenetic diversity, Shannon’s entropy and Pielou’s evenness, and statistical significance was assessed using the Kruskal–Wallis tests. Beta diversity was assessed by weighted and unweighted UniFrac metrics and visualized by Principal Coordinates Analysis (PCoA), with statistical significance assessed with Permutational Multivariate Analysis of Variance (PERMANOVA) tests, with 999 permutations.

## Results

DNA sequencing of all 203 samples resulted in 13,691,505 reads with a mean of 60,315, which underwent read-pair joining and quality filtering to 10,789,538 reads with a mean of 47,531. All samples were denoised into 9,918 Amplicon Sequence Variants (ASVs); when wild samples were filtered (*n* = 159), they had a total of 9,675 ASVs, and when captive samples were filtered (*n* = 44), they had a total of 1,815 ASVs, resulting in only 243 shared ASVs between wild and captive samples.

### Characterising the Wild Echidna Gut Microbiome

**Figure 2:**
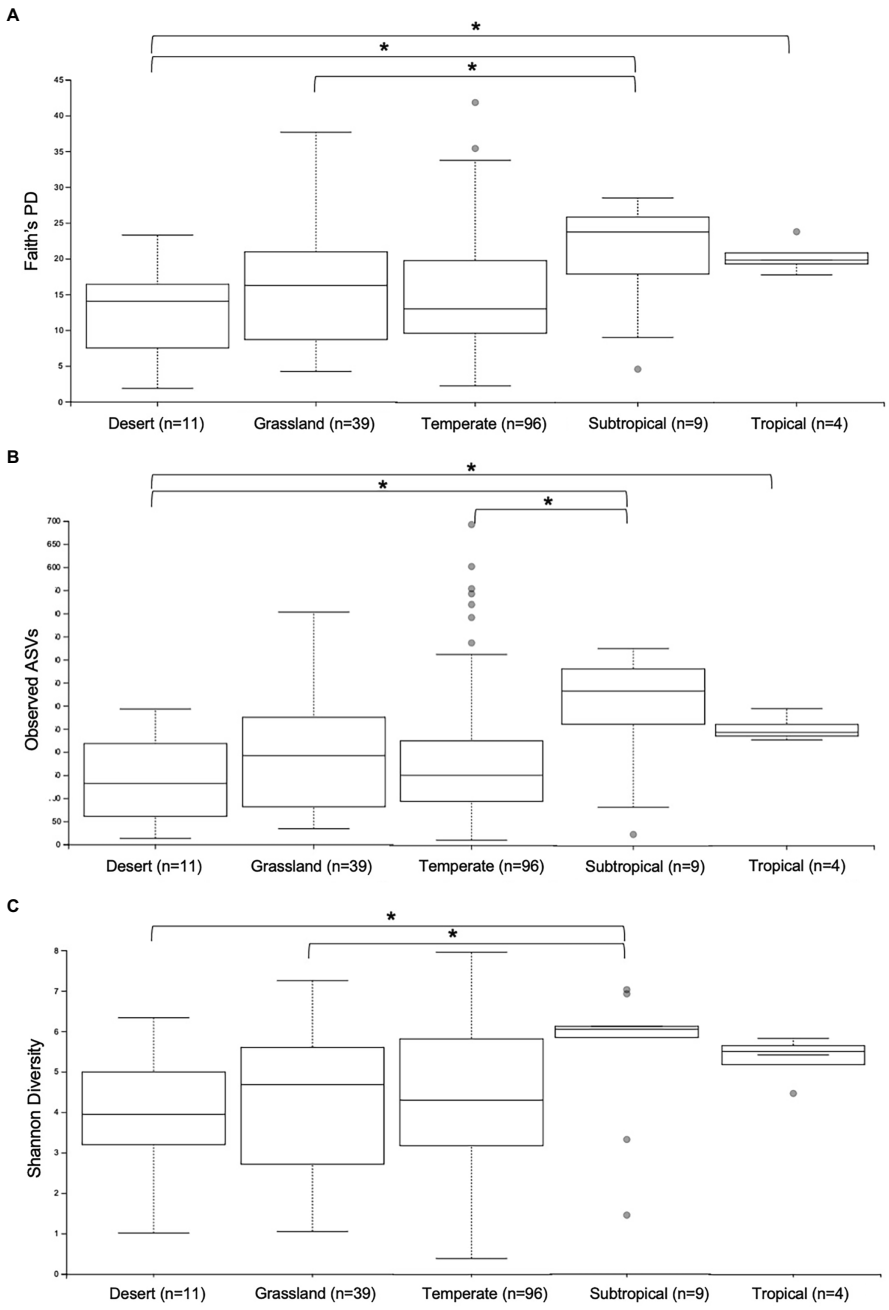
Alpha diversity analyses of gut microbiomes from wild samples collected in different climate regions across Australia. Whisker-box plots depict the following metrics: **(A)** Faith’s phylogenetic diversity (Faith’s PD); **(B)** Observed Amplicon Sequence Variants; **(C)** Shannon’s Diversity index. Horizontal lines indicate median values, upper and lower bounds represent the 25th and 75th percentiles, and top and bottom whiskers indicate maximum and minimum values. Outliers are shown as grey circles. ^*^= 0.01 < *p* < 0.05.

First, the gut microbiomes of wild echidna samples were analysed, revealing extraordinary variability of the individual samples. We endeavoured to correlate this variation with climate, vegetation, land-use, and seasonal aspects based on the locations each scat was collected (Supplementary Table S1). Analysis of alpha diversity revealed significant differences in samples collected in areas with differing climate; typically, samples collected from subtropical and tropical regions had greater number of ASVs (observed ASVs), greater phylogenetic diversity (Faith’s phylogenetic diversity) and greater richness (Shannon’s diversity) than samples collected in desert, grassland and temperate locations (Supplementary Table S4; Figure 2). Due to limited number of samples from tropical regions (*n* = 4), these results should be tested in the future with greater sample size.

Next, we investigated microbial composition and saw significant differences in beta diversity (unweighted UniFrac) influenced mostly by seasonal changes (*p* = 0.001) and anthropogenic biomes (*p* = 0.013), which describes how the land is used by people (i.e. croplands, rangelands, urbanised; Supplementary Tables S1 and S5). Land-cover was also considered statistically different between some groups; for example, samples from native grasslands (*n* = 27) had significantly different microbial communities to samples collected from land used for annual crops (*n* = 34; *p* = 0.011; Supplementary Table S5). Although we endeavoured to correlate the wild samples’ microbial diversity to several types of location metadata, no single metric could explain the differences in taxonomic communities present in the echidna scats (see PCoA plots in Supplementary Figure S2 and interactive PCoA in Supplementary File 1).

**Figure 3:**
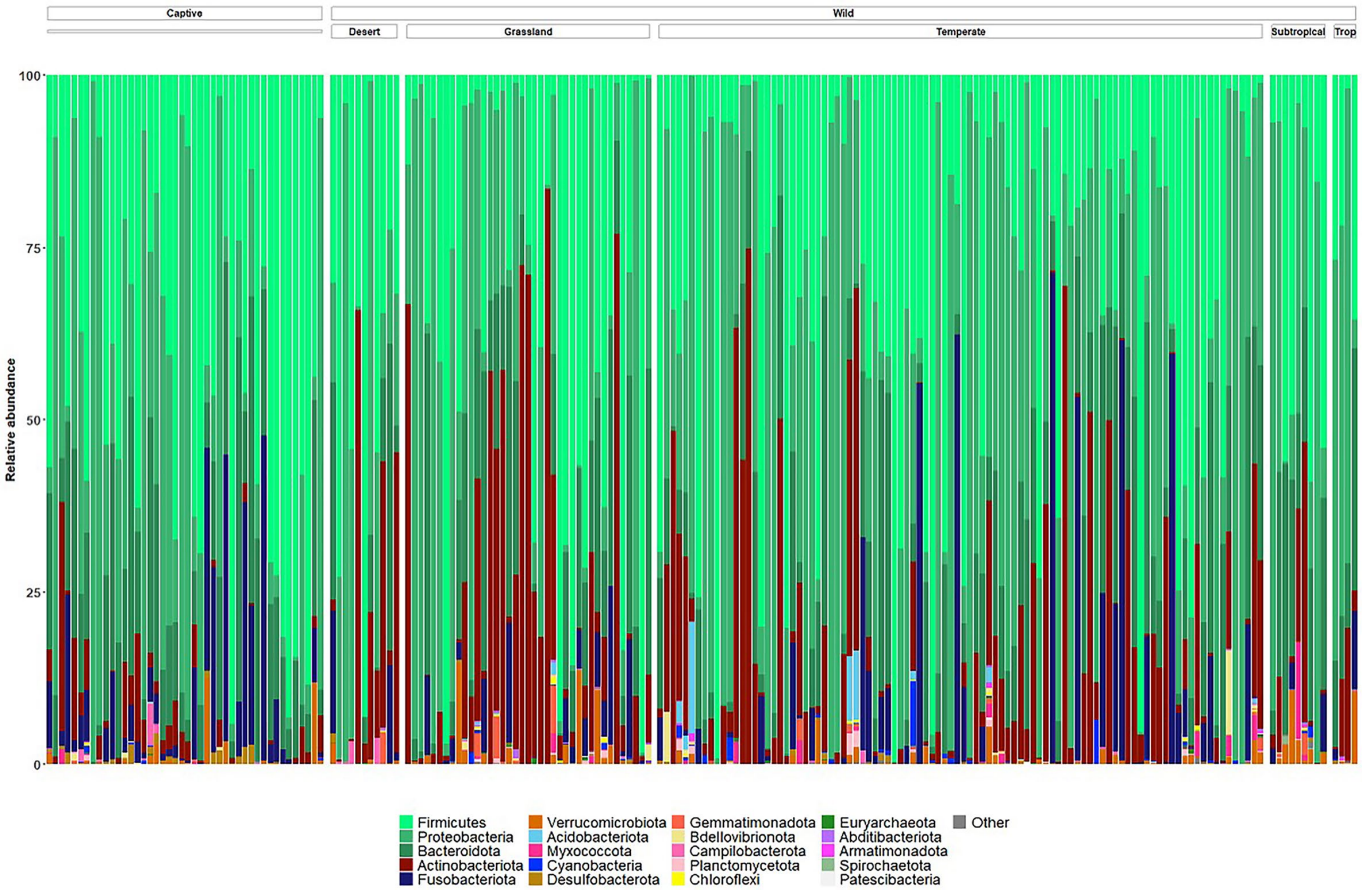
Taxonomy bar plots of relative frequency of bacteria present in all wild and captive echidna scats at the phylum level. Samples are firstly organised into ‘captive’ and ‘wild’ samples, and the wild samples are further organised by climate class (Supplementary Table S1). Top 20 most abundant phyla are coloured in figure; legend is labelled with most abundant taxa on the left to least abundant taxa on the right. Original and interactive QZV files to view bar plots are available in Supplementary Files.

The microbiomes of wild echidna scat samples were dominated by bacteria from phyla Proteobacteria, Firmicutes, Bacteroidota, Actinobacteriota, and Fusobacteriota, with some samples also containing low abundances of Verrucomicrobiota, Myxococcota, Cyanobacteria, Acidobacteriota and even lower abundances of several other phyla (Figure 3; Supplementary File 2). Despite an overall high variability of the bacteria seen between wild samples (*n* = 159), the most prevalent bacteria were from the following genera (numbers in square brackets represent number of wild samples with the bacteria present): *Arthrobacter* [140], *Enterococcus* [119], *Enterobacteriaceae* [111], *Escherichia-Shigella* [102], *Lactococcus* [98], *Fusobacterium* [95], *Bacillus* [94], *Romboutsi*a [92], *Pediococcus* [79], *Paeniclostridium* [61], *Acinetobacter* [63], and *Sanguibacter* [59], and *Pseudomonas* [50] (Supplementary Figure S3); no bacterial taxon was found in all samples.

### Captivity Significantly Changes the Gut Microbiomes in Comparison With Wild Echidnas

**Figure 4:**
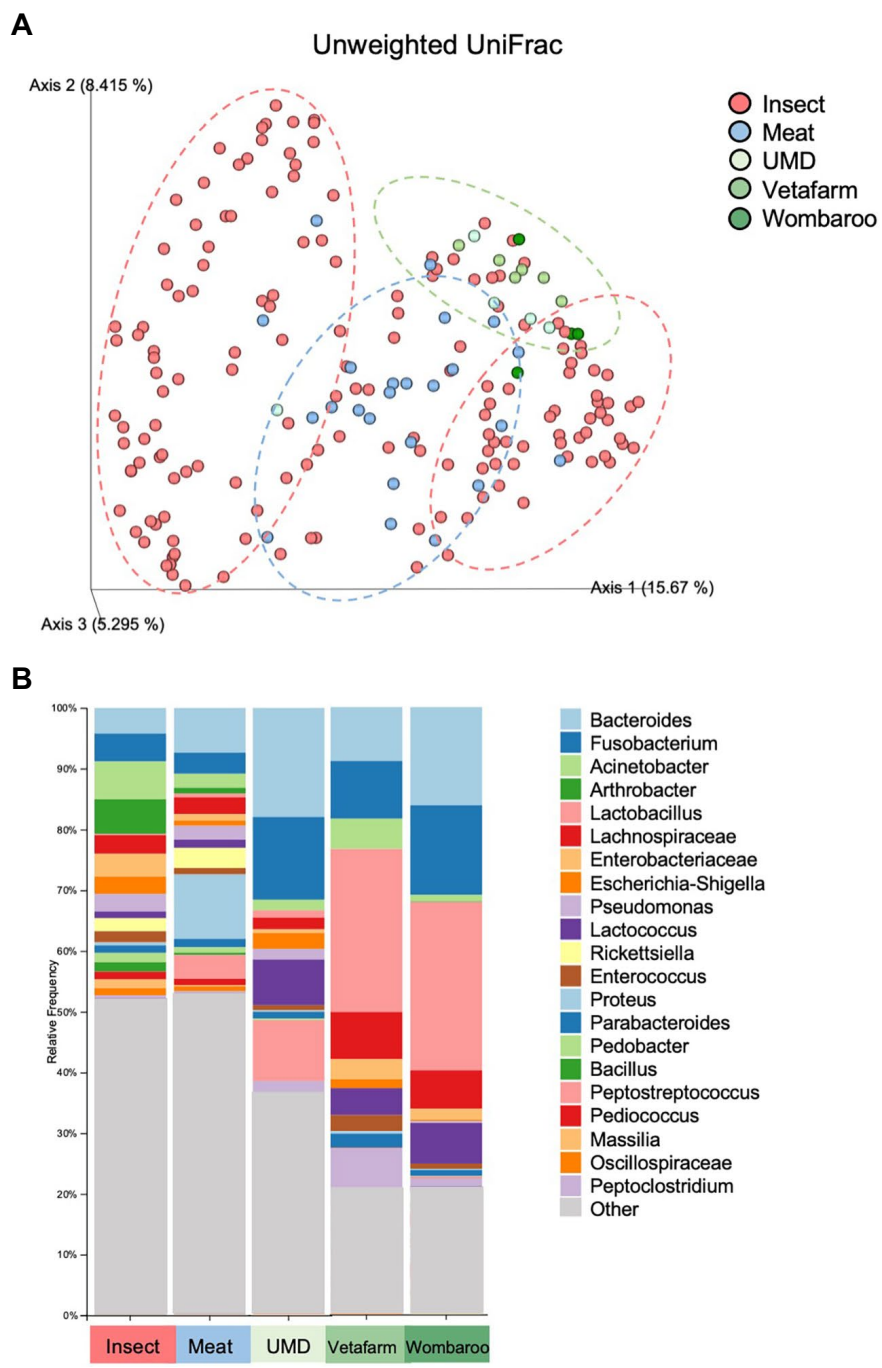
Differences in microbial composition are observed between samples collected in wild compared to samples collected in captivity from echidnas fed four different diets. **(A)** PCoA plot of unweighted UniFrac distances showing clustering patterns of wild samples (insect diet) and zoo samples (Meat, UMD, Vetafarm and Wombaroo diets). Green dotted circle shows the Taronga Zoo samples mostly clustering together; Blue dotted circle shows the Perth Zoo samples mostly clustering together between the two major clusters of the Wild samples within the red dotted circles. **(B)** Taxonomy bar plots showing relative frequencies of bacteria present in echidna scats shown at the genus or family level; all samples have been aggregated according to their diet, and an average relative frequency is shown. Samples are labelled by their diet where insect refers to wild collected samples. UMD, Updated Meat Diet. Original and interactive QZV files to view bar plots are available in Supplementary Files.

Captive animals often feature different microbiomes when compared to wild conspecifics. We assessed this in echidnas and in addition investigated if different diets had an effect on their microbiomes. There were no differences observed for alpha diversity metrics (*p* > 0.05; Faith’s PD, Observed ASVs, Shannon Diversity) between samples collected in the wild and in zoos (Supplementary Table S6; Supplementary Figure S4). However, the two groups (wild vs. captive) were significantly different in regard to microbial composition (unweighted UniFrac distances *p* = 0.001; Supplementary Table S7; Figure 4A; Supplementary File 3). Both wild and zoo-held echidnas shared most of the same abundant bacteria phyla (Firmicutes, Proteobacteria, Bacteroidota, Actinobacteriota, and Fusobacteriota); however, there was very little overlap in the most abundant genera observed between the two groups. For example, echidnas fed captive diets had abundances of *Bacteroides*, *Proteus*, *Lactobacillus*, *Peptostreptococcus*, *Lactococcus*, uncharacterised *Lachnospiraceae*, and *Peptoniphilus*. Very small abundances of *Acinetobacter* were observed, which was one of the most prevalent bacteria in the wild samples. *Bacteroides* and *Fusobacterium* were the only prominent bacteria in the zoo samples that was seen frequently in wild samples (Figure 4B; Supplementary File 4).

### Different Diets Fed in Captivity Affect the Echidnas’ Gut Microbiomes

**Figure 5:**
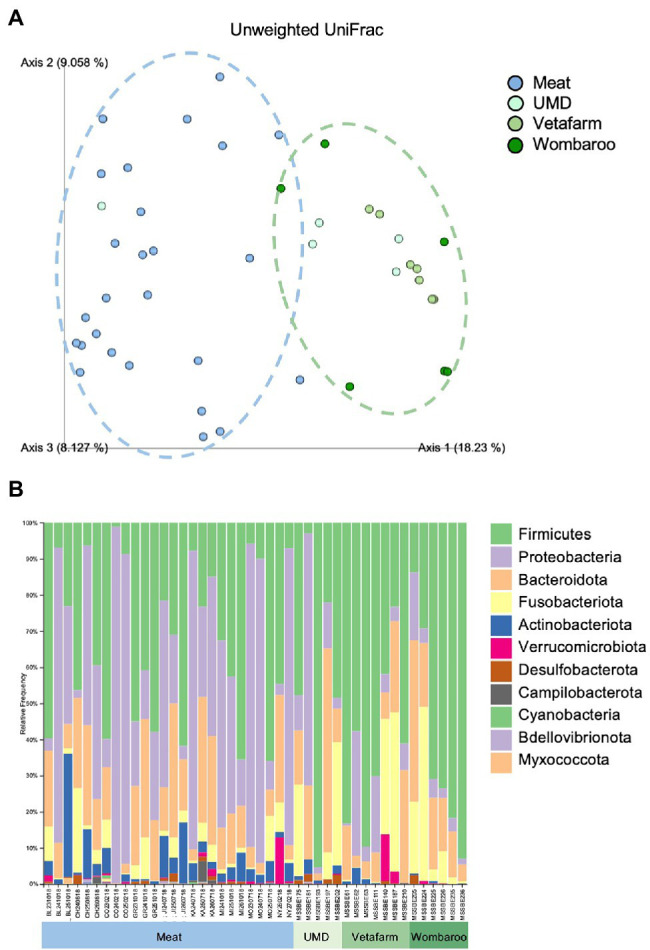
Differences in microbial composition are observed between Meat diet and all other diets fed in captivity. **(A)** PCoA plot of unweighted UniFrac distances showing separation between Meat diet clustering further to the left of Axis 1 (blue dotted circle) and other diets clustering to the right of Axis 1 (green dotted circle). **(B)** Taxonomy bar plots showing relative frequencies of bacteria present in echidna scats shown at the phylum level. Samples are labelled by their sample ID and diet (Supplementary Figure S2). Most prevalent phyla are included in the legend; as bar colours repeat, the legend is labelled with most abundant taxa on top to least abundant taxa on bottom of legend. UMD, Updated Meat Diet. Original and interactive QZV files to view bar plots are available in Supplementary Files.

Lastly, we assessed how different diets in captivity may affect the microbiomes of echidnas. Of the four diets tested, we found that the gut microbiome from echidnas fed the Meat diet was more phylogenetically diverse than all other diets, had greater number of ASVs than UMD and Vetafarm, and had a greater Shannon’s diversity when compared to the Vetafarm diet (*p* < 0.05; Supplementary Table S8; Supplementary Figure S5). There were no statistically significant alpha diversity differences observed between UMD, Vetafarm and Wombaroo diets (*p* > 0.05; Supplementary Table S8). Unweighted UniFrac distances also showed significant differences in microbial composition between Meat and all other diets (*p* = 0.001; Supplementary Table S9; Figure 5A; Supplementary File 5).

Similar to the samples collected from wild echidnas, the major phyla present in samples collected from captive echidnas include Firmicutes, Proteobacteria, Bacteroidota, Fusobacteriota, and Actinobacteriota, with lower abundances of Verrucomicrobiota, Desulfobacterota, Campilobacterota, Bdellovibrionota, Cyanobacteria, and Myxococcota. Several genera were observed in most or all zoo samples including *Bacteroides*, *Fusobacterium*, *Lactococcus*, *Acinetobacter*, *Parabacteroides*, *Enterococcus*, *Erysipelatoclostridium*, *Escherichia-Shigella*, and uncharacterised genera of *Enterobacteriaceae* family. *Rickettsiella* and *Peptoniphilus* were only present in samples from echidnas fed the Meat diet. *Pseudomonas* mostly appeared in Meat diet, and *Proteus* was more abundant in Meat diet. *Peptostreptococcus* was found commonly in echidnas fed the Meat or Updated Meat Diet, while *Lactobacillus* and *Lachnospiraceae* were found exclusively in samples from echidnas fed the Vetafarm and Wombaroo diets (Supplementary Figure S3). For echidnas held at Perth Zoo where samples were taken across three consecutive days, daily variation in microbial composition was observed (Figure 5B; Supplementary Figure S3). Technical triplicates from two samples confirm that this is not a result of technical variation (Supplementary Figure S6).

## Discussion

This study is the first characterisation of the short-beaked echidna gut microbiome and the largest geographical microbiome study of any native Australian mammal. We show here that the major phyla forming the gut microbiome consist of Proteobacteria, Firmicutes, Bacteroidota, Actinobacteriota, and Fusobacteriota. These are consistent with gut microbiota in most mammals, where Firmicutes and Bacteroidota are usually the dominant groups (Ley et al., 2008). Wild echidnas have a much greater abundance of Proteobacteria, which has often been associated with gut dysbiosis (Shin et al., 2015). However, in echidnas, this is due to the dominating genus *Acinetobacter* (which belongs to Proteobacteria phylum); *Acinetobacter* is a common soil bacterium (Acer et al., 2020), which is consistent with echidnas consuming large amounts of soil when foraging. This is further supported by the presence of *Arthrobacter*, another prolific soil bacterium (Radkov et al., 2016) and the second most abundant bacteria genus in wild samples. Interestingly, along with soil and environmental bacteria, the next most abundant groups were plant-fermenting and lactic acid bacteria, including *Lachnospiraceae, Pedicococcus, Enterococcus*, *Lactococcus*, and *Oscillospiraceae* (Teuber, 1995; Biddle et al., 2013; Porto et al., 2017; George et al., 2018). This suggests that plant material may be a much more prominent part of the echidna diet than has been previously recognised. It also raises the question whether the echidna gut system can be considered fermentative with the combination of an abundance of these putatively fibre-fermenting bacteria and a monogastric digestive tract, which is how hindgut-fermenting mammals, such as odd-toed ungulates (horses and rhinoceroses), rodents, rabbits and koalas, digest cellulose (Prins and Kreulen, 1990).

A large proportion of *Fusobacterium* are not commonly seen in mammal gut microbiomes (Ley et al., 2008); however, some wild echidna samples had up to 70% relative abundance of this bacterium. In humans, some species of *Fusobacterium* can be attributed to colorectal cancer (Castellarin et al., 2012), and other diseases (Han, 2015), but it is rare to find it in faecal samples. It has, however, been observed in the proximal and distal intestines of Atlantic cod (Zhou et al., 2013), large intestine of vultures (Roggenbuck et al., 2014) and more recently in faecal material collected from the rectum of jackals (Menke et al., 2017), with no evidence of being pathogenic. Furthermore, as *Fusobacterium* was also present in the zoo echidna samples, it may be a gut commensal in echidnas as these animals were considered healthy at time of sampling.

This work also revealed the presence of *Rickettsiella* in wild samples collected in South Australia, New South Wales, and Victoria. This bacterium is associated with hard ticks and can be pathogenic to both the tick hosts and to mammals if transmitted (Leclerque and Kleespies, 2012). Echidnas can often be infested with hard ticks, and there is even a species of tick that is recognised to live almost exclusively on echidnas (*Bothriocroton concolor*; Roberts, 1970), although this species has also been observed on Kangaroo Island kangaroos (Oorebeek and Rismiller, 2007). In three samples (located in Kangaroo Island and Waitpinga in SA, and Wamboin in NSW), *Rickettsiella* was 80–90% of total bacterial abundance indicating echidnas may be frequently ingesting ticks, which has been observed in the wild (P. Rismiller, Pers. Comms., Nov 2020).

Our finding of significant changes in gut microbiome of captive echidnas has been observed in different mammalian taxa (McKenzie et al., 2017) and is likely due to different diets and environments. A similar dramatic shift was observed in the aardvark and giant ant eater, which are ant and termite eating species, which also have nutrition-related health problems due to the difficulty in creating appropriate diets (Clark et al., 2016; McKenzie et al., 2017). Rather than soil and environmental bacteria forming the majority of the microbiome as is seen in wild echidnas, zoo-held echidnas had a greater proportion of Bacteroidota, especially *Bacteroides* and *Parabacteroides*, which are common gut commensals (Hiippala et al., 2020). Interestingly, although captive echidnas are fed a carnivorous-modelled diet with meat as the main ingredient, there were still high proportions of putative plant-fermenting and lactic acid bacteria present in their gut, suggesting that echidnas may naturally be herbivorous hindgut fermenters. In 2017, Shaw suggested that echidnas be reclassified as insectivorous herbivores, which is supported by our findings (M. Shaw, Pers. Comms., Nov 2020).

Diet appears to play a significant role in the formation of the echidna gut microbiome. Even subtle differences in diets fed in zoos resulted in microbial community and diversity changes, particularly when comparing the Meat diet to the three other diets fed at Taronga Zoo. Location effects, such as water source or soil in enclosure, may also enhance these differences, as the Meat diet was exclusively fed at Perth Zoo and showed the greatest dissimilarity. The Meat diet shows greater microbial diversity but also potentially pathogenic bacteria, such as *Proteus* (a pathogen found in beef mince; Doulgeraki et al., 2011) and *Rickettsiella*. Whereas in the diets fed to echidnas in Taronga Zoo, there were greater abundances of gut commensals and even *Lactobacillus*; however, this may be coming directly from the food source as the Vetafarm and Wombaroo diets include a dry yeast probiotic that may contain *Lactobacillus*. Both zoo populations were healthy at the time of sampling and reproduced successfully (Ferguson and Turner, 2013; Perry et al., 2019); therefore, future research is needed to understand the relationships of gut bacteria to echidna health specifically, as ‘pathogenic’ and ‘probiotic’ associations of bacteria are concluded mostly from human and mouse studies.

Another feature of the echidna gut microbiome is how variable it can be. In wild echidnas, not a single bacteria genus was shared across all samples. Even in captivity, where echidnas are housed in the same environment and provided the same food, there were (sometimes major) differences observed in bacterial communities and relative abundances within individuals from samples collected across three consecutive days. This daily variation, in combination with diet heavily influencing the gut microbiome, may explain the large variability observed in the wild scats and how multiple samples collected from the same location contained different bacterial profiles. As echidnas will opportunistically forage throughout the day, often travelling large distances, their daily gut microbiomes will likely depend on what food is available and in what environments they forage in. It would be ideal to examine the diet contents in these scats (either physically or genetically) to determine if these correlations exist.

This study investigates, for the first time, the microbial diversity and composition in the echidna gut; the most widespread native Australian mammal. We find that diet plays a major role in defining the echidna gut microbiome, with striking differences observed between the wild samples and those from echidnas kept in captivity. Collaboration with the Australian community *via* the EchidnaCSI project enabled sampling across Australia. This revealed an enormous amount of variation in gut microbiota in wild echidnas. Some common gut commensals were present in both wild and zoo samples; however, most of the gut bacteria observed were soil or plant associated and large abundances of Actinobacteria and Fusobacteria, which appear unique to echidnas as they are not common in other mammals. It will be important in the future to determine the pathology and probiotic relationships between the bacteria and echidnas in order to make meaningful connections to echidna health and to determine if we should be concerned with the differences observed in managed echidnas. Furthermore, a powerful way of assessing whether diet is the driving factor behind the wild microbial diversity will be to generate genetic profiles of echidna diet from scats to correlate with microbiome data. This research has provided new insights and first steps towards understanding gut microbiota of an iconic Australian mammal.

## Data Availability Statement

The datasets presented in this study can be found in online repositories. The names of the repository/repositories and accession number(s) can be found at: NCBI PRJNA811647.

## Author Contributions

TP performed sample processing, laboratory work, data analysis, created figures, and wrote the manuscript. EW performed laboratory work, data analysis, and co-wrote sections of the manuscript. RE provided guidance and resources for the microbiome sequencing methods and aided in interpretation and visualisation of data. AS created the EchidnaCSI app which enabled sample collection. IW performed sample processing and data management. BL collected samples and provided guidance on echidna biology. PR and MS collected samples, guided the design of the project, aided in interpretation of results, and edited manuscript. FG contributed to design and supervision of the project as well as manuscript preparation and evaluation. All authors contributed to the article and approved the submitted version.

## Funding

This research was funded by the Environment Institute at The University of Adelaide. TP and AS are funded by the Research Training Program. FG is an Australian Research Council Future Fellow. PR acknowledges the Pelican Lagoon Research Centre for research support and resources. Funding sources had no involvement in study design; in the collection, analysis and interpretation of data; in the writing of the report; or in the decision to submit the article for publication.

## Conflict of Interest

The authors declare that the research was conducted in the absence of any commercial or financial relationships that could be construed as a potential conflict of interest.

## Publisher’s Note

All claims expressed in this article are solely those of the authors and do not necessarily represent those of their affiliated organizations, or those of the publisher, the editors and the reviewers. Any product that may be evaluated in this article, or claim that may be made by its manufacturer, is not guaranteed or endorsed by the publisher.

## Footnotes

^1^ https://spatial.ala.org.au/layers
