# Supplementary material for: Characterising the Gut Microbiomes in Wild and Captive Short-Beaked Echidnas Reveals Diet-Associated Changes
Source: Front Microbiol. 2022 Jun 30;13:687115. doi: 10.3389/fmicb.2022.687115 (PMC9279566; doi:10.3389/fmicb.2022.687115)
Supplement: Supplementary file 2 [file Data_Sheet_2.docx]

**Supplementary Figures**

**
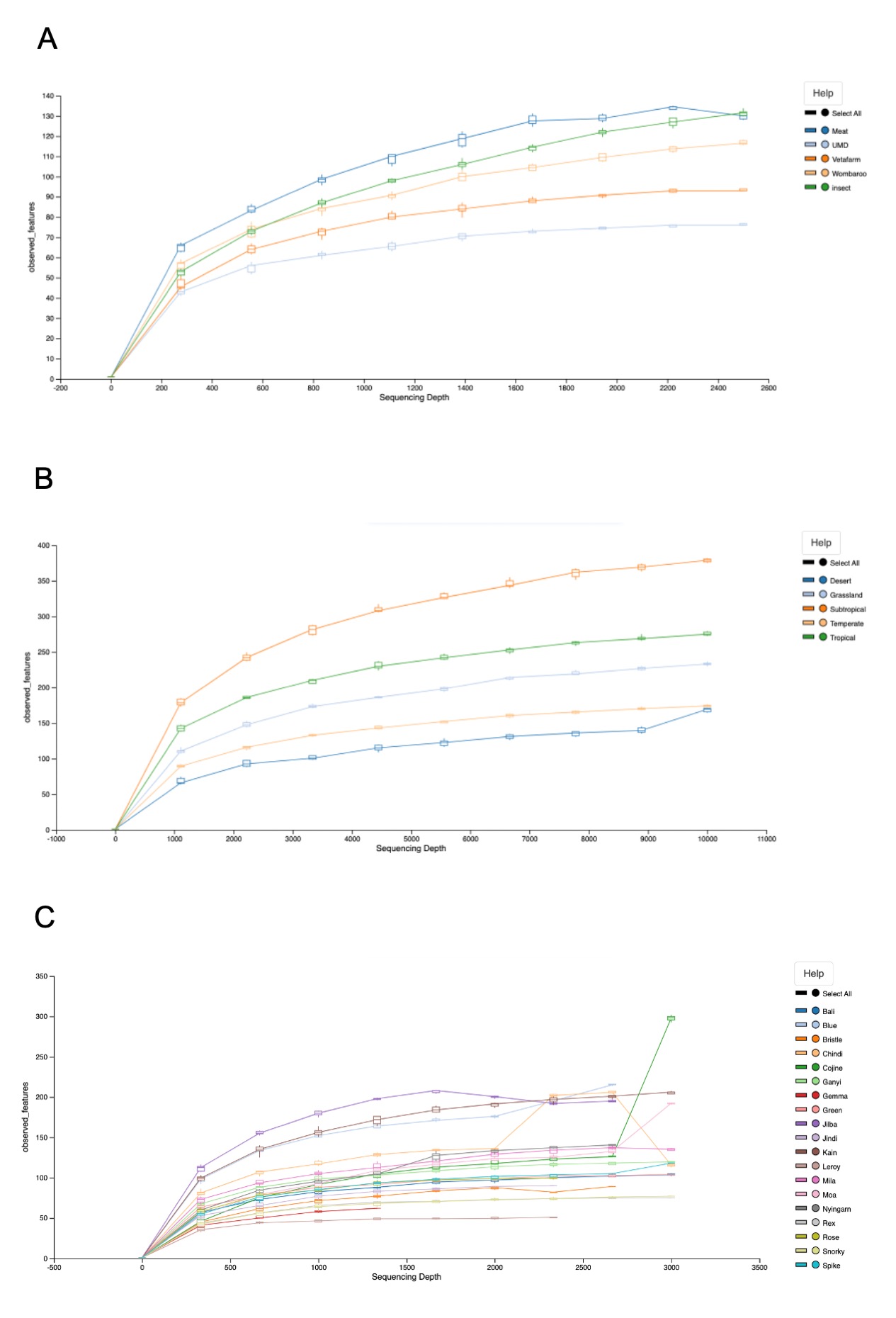
**

**Figure S1:** **Alpha rarefaction curves.** A) All samples (both captive and wild); B) Wild samples only; C) Captive samples only.

**
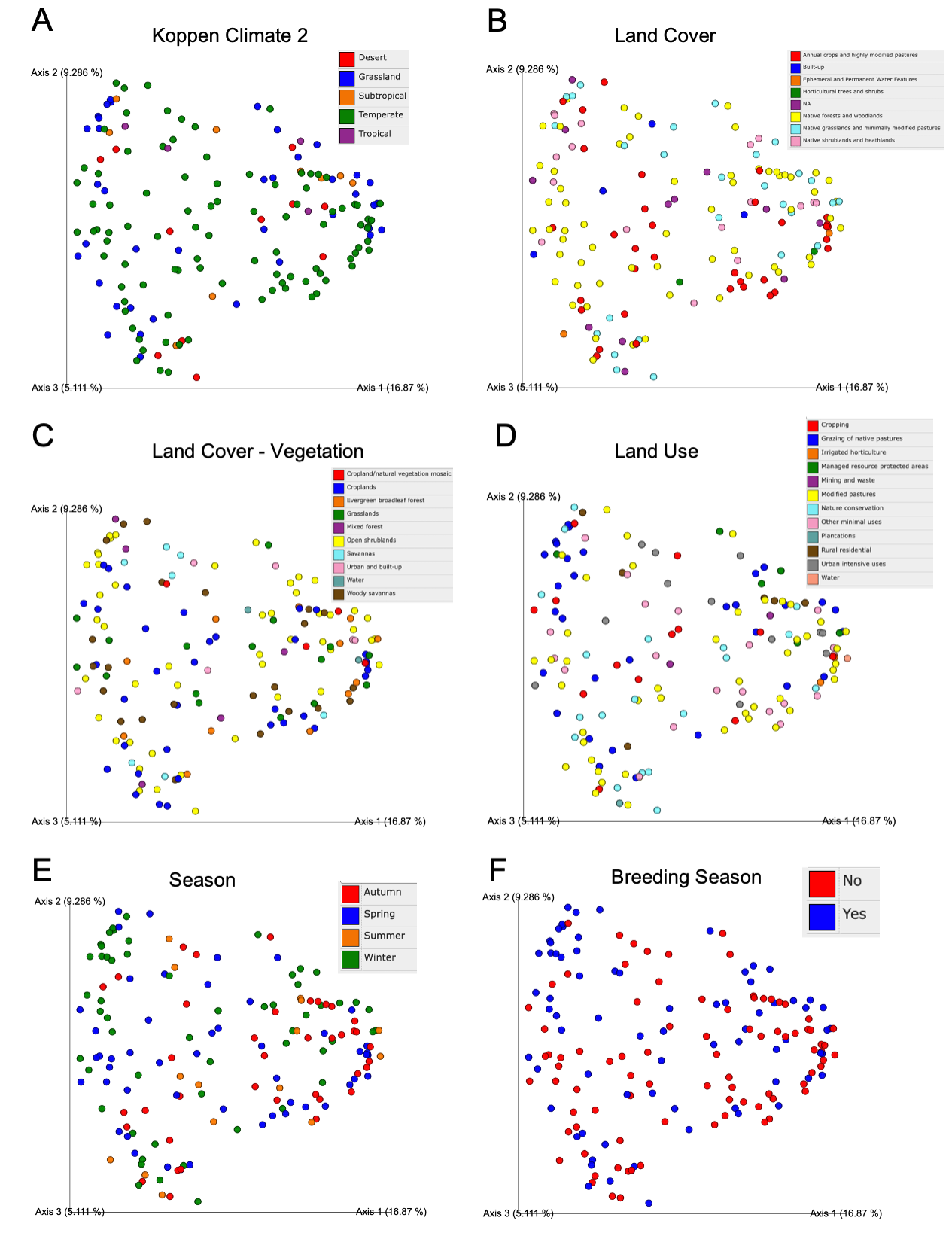
**

**Figure S2: Unweighted UniFrac PCoA plots for wild echidna samples.** Each plot has been coloured according to a different location-based metadata (Table 1). Showing here that no single factor could explain the microbial community structure of the wild samples.


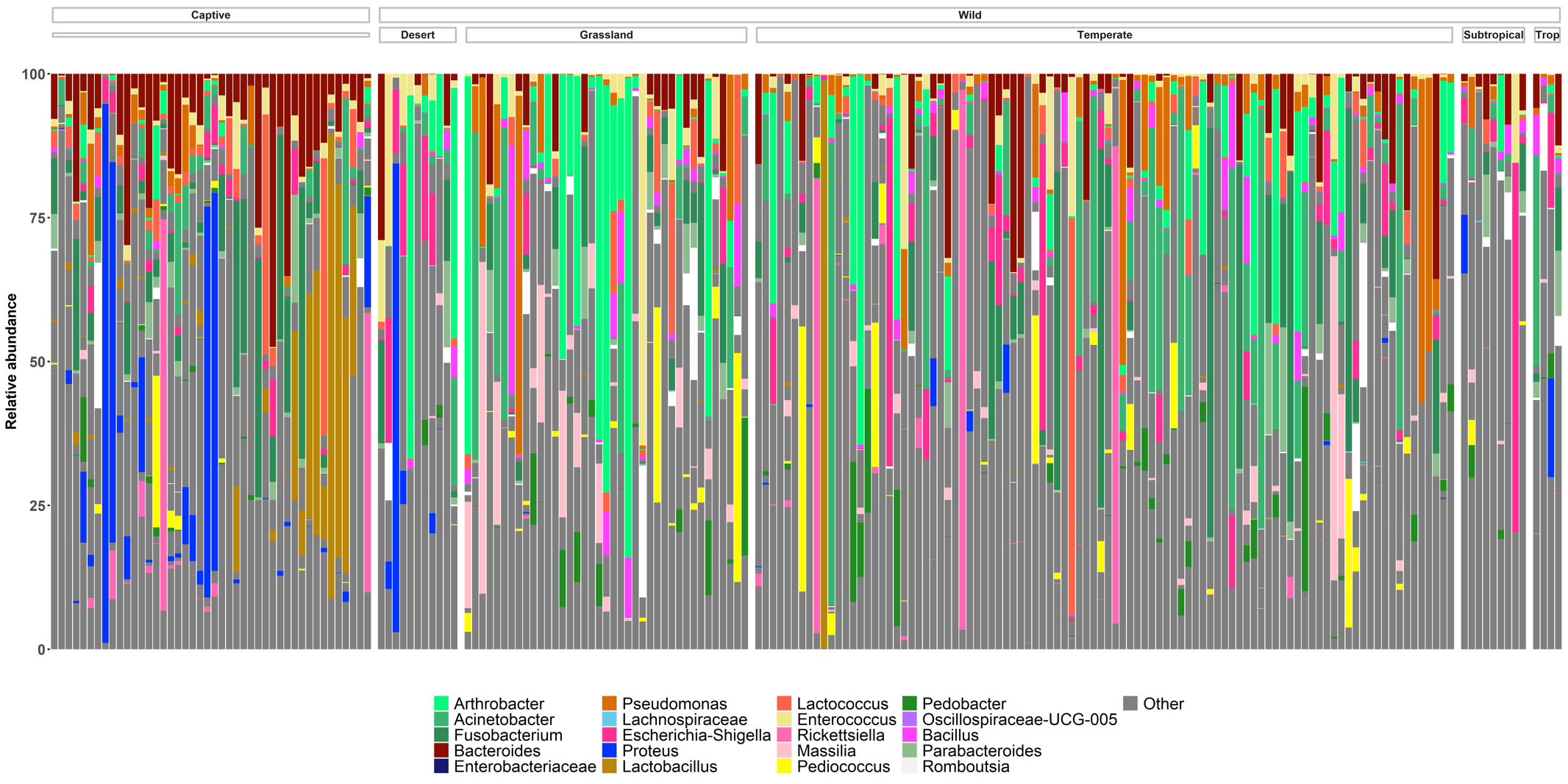


**Figure S3:** **Taxonomy bar plots of relative frequency of bacteria present in all captive and wild echidna scats at the genus level.** Samples are firstly organised into ‘captive’ and ‘wild’ samples, and the wild samples are further organised by climate class (Table 1). The top 20 genera present are visualised by coloured bars and included in the legend; all other taxa are coloured grey and labelled as ‘other’. Legend is labelled with most abundant taxa on the left to least abundant taxa on the right. To visualise all taxa present in the samples, the interactive QZV file is available to view in supplementary material.


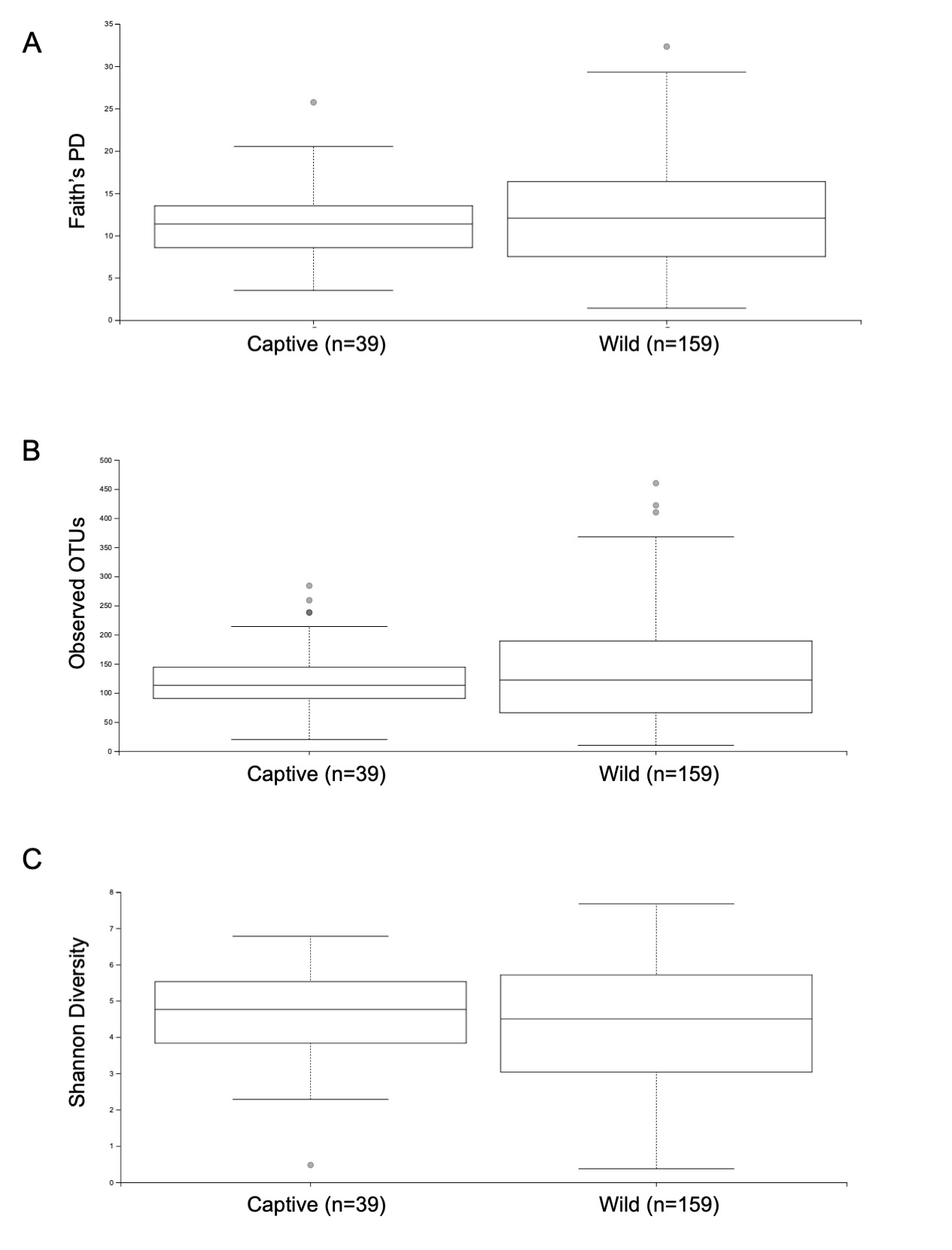


**Figure S4:** **Alpha diversity analyses of gut microbiomes from samples collected in captivity compared to those collected in the wild.** Whisker-box plots depict the following metrics: A) Faith’s phylogenetic diversity (Faith’s PD); B) Observed ASVs; C) Shannon’s Diversity index (captive). Horizontal lines indicate median values, upper and lower bounds represent the 25th and 75th percentiles, and top and bottom whiskers indicate maximum and minimum values. Outliers are shown as grey circles. No significance was observed for any of these diversity metrics. No significance was observed between these two groups for any of the above metrics (Table S4).

**
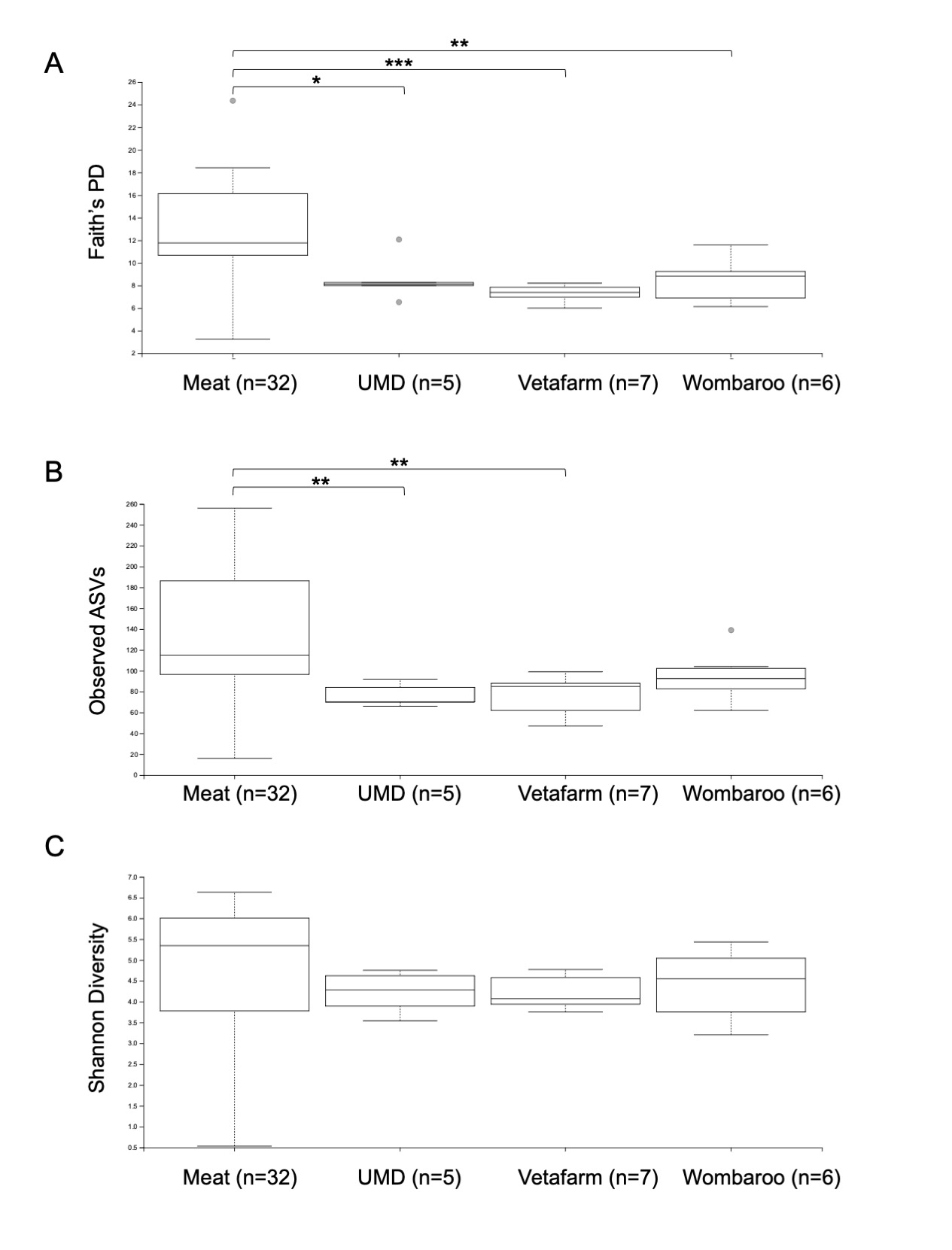
**

**Figure S5: Alpha diversity analyses of gut microbiomes from samples collected in captivity of echidnas fed four different diets.** Whisker-box plots depict the following metrics: A) Faith’s phylogenetic diversity (Faith’s PD); B) Observed ASVs; C) Shannon’s Diversity index. Horizontal lines indicate median values, upper and lower bounds represent the 25th and 75th percentiles, and top and bottom whiskers indicate maximum and minimum values. Outliers are shown as grey circles.

* = p<0.05, ** = p<0.01, *** = p<0.001; UMD = Updated Meat Diet.

**
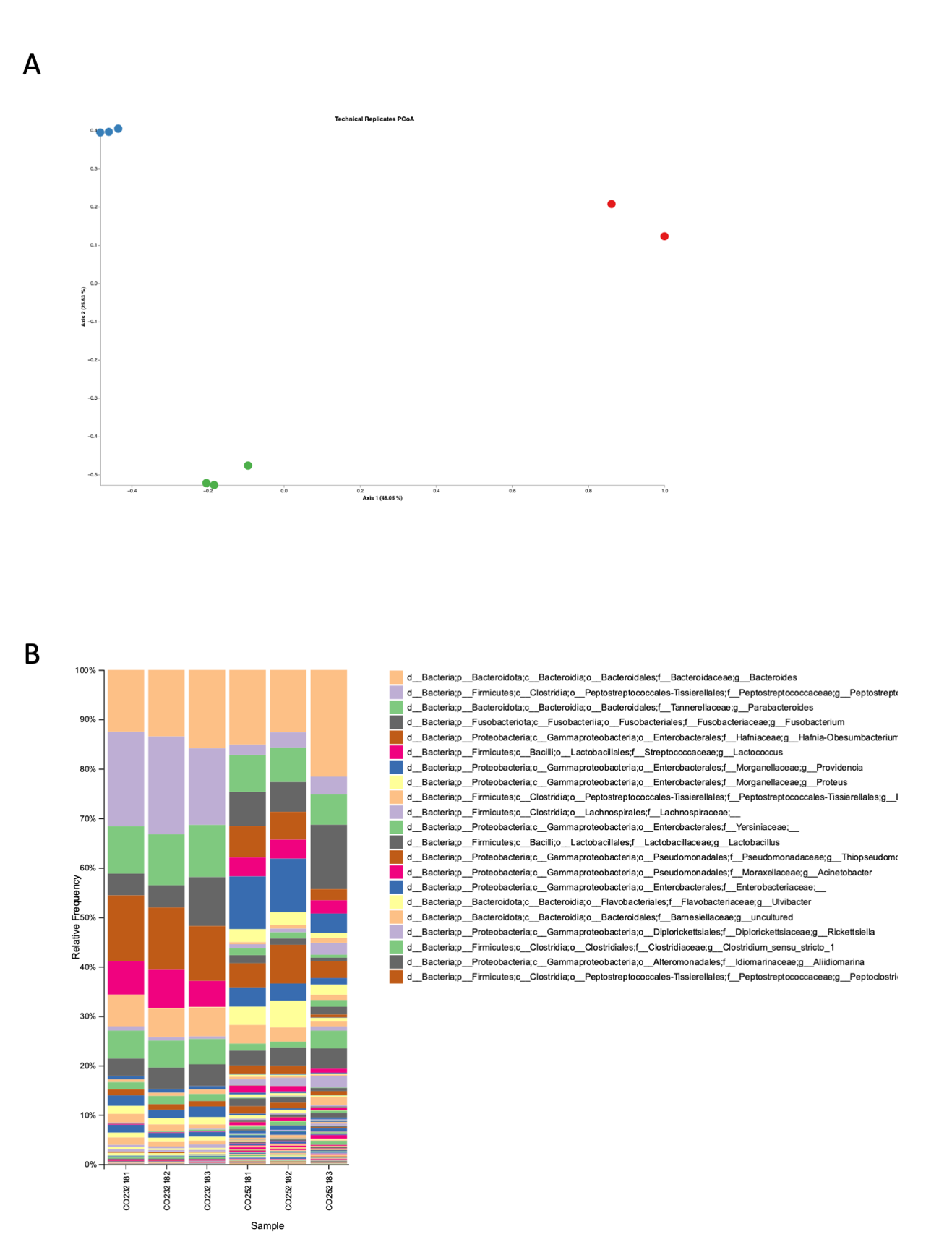
 Figure S6: Technical triplicates confirm daily variation is true biological phenomenon and not due to technical issues.** A) PCoA plot of unweighted UniFrac distances showing tight clustering of triplicates extracted from two samples collected two days apart from the same echidna: CO23218 (blue) and CO25218 (green); red samples indicate negative controls (no template 16S PCRs that were sequenced to capture potential contamination). B) Taxonomy bar plots showing relative frequencies of bacteria present technical triplicates at the genus and family level. Samples are labelled by their sample ID (Table 2). The top 21 genera and families present are included in the legend, however only the most abundant are easily visualised; as bar colours repeat, the legend is labelled with most abundant taxa on top to least abundant taxa on bottom.
