# Supplementary material for: Characterising the Gut Microbiomes in Wild and Captive Short-Beaked Echidnas Reveals Diet-Associated Changes
Source: Front Microbiol. 2022 Jun 30;13:687115. doi: 10.3389/fmicb.2022.687115 (PMC9279566; doi:10.3389/fmicb.2022.687115)
Supplement: Supplementary file 3 [file Data_Sheet_3.docx]

**Supplementary Tables**

**Table S1: Information for faecal samples collected from wild echidnas.** Location = State followed by suburb sample was collected in; DOC = date of collection.

| Sample ID | Latitude | Longitude | Location | Season | Breeding season | DOC | Land use | Anthropogenic biomes | Climate class | Land cover |
| --- | --- | --- | --- | --- | --- | --- | --- | --- | --- | --- |
| 170901SA1 | -33.988 | 138.614 | SA;Watervale | Spring | Yes | 1/9/17 | Cropping | Populated croplands | Temperate | Annual crops and highly modified pastures |
| 170916SA1 | -36.970 | 140.370 | SA;Lucindale | Spring | Yes | 16/9/17 | Urban intensive uses | Populated croplands | Temperate | Built-up |
| 170917SA1 | -35.180 | 138.790 | SA;Meadows | Spring | Yes | 17/9/17 | Modified pastures | Residential rainfed croplands | Temperate | Annual crops and highly modified pastures |
| 170917VIC1 | -37.689 | 143.997 | VIC;Lal Lal | Spring | Yes | 17/9/17 | Rural residential | Populated croplands | Temperate | Native forests and woodlands |
| 170920SA1* | -35.750 | 137.690 | SA;Kangaroo Island | Spring | Yes | 20/9/17 | Cropping | Populated croplands | Temperate |  |
| 170920SA2 | -35.067 | 138.915 | SA;Mount Barker | Spring | Yes | 20/9/17 | Modified pastures | Mixed settlements | Temperate | Annual crops and highly modified pastures |
| 170927SA1* | -35.740 | 137.640 | SA;Kangaroo Island | Spring | Yes | 27/9/17 | Modified pastures | Populated croplands | Temperate |  |
| 170928SA2 | -34.880 | 138.720 | SA;Montacute | Spring | Yes | 28/9/17 | Nature conservation | Urban | Temperate | Native forests and woodlands |
| 171001SA4 | -30.451 | 139.090 | SA;Gammon Ranges | Spring | No | 1/10/17 | Nature conservation | Remote rangelands | Grassland | Native forests and woodlands |
| 171002SA1* | -35.600 | 137.580 | SA;Kangaroo Island | Spring | No | 2/10/17 | Cropping | Populated croplands | Temperate |  |
| 171007SA1* | -35.740 | 137.640 | SA;Kangaroo Island | Spring | No | 7/10/17 | Modified pastures | Populated croplands | Temperate |  |
| 171008SA4* | -35.740 | 137.640 | SA;Kangaroo Island | Spring | No | 8/10/17 | Modified pastures | Populated croplands | Temperate |  |
| 171011SA1* | -35.750 | 137.690 | SA;Kangaroo Island | Spring | No | 11/10/17 | Cropping | Populated croplands | Temperate |  |
| 171012SA1 | -34.901 | 138.772 | SA;Montacute | Spring | No | 12/10/17 | Urban intensive uses | Residential woodlands | Temperate | Native grasslands and minimally modified pastures |
| 171101NSW1 | -34.618 | 149.708 | NSW;Middle Arm | Spring | No | 1/11/17 | Modified pastures | Populated rangelands | Temperate | Native forests and woodlands |
| 171101NSW2 | -34.618 | 149.708 | NSW;Middle Arm | Spring | No | 1/11/17 | Modified pastures | Populated rangelands | Temperate | Native forests and woodlands |
| 171101NSW3 | -34.422 | 150.139 | NSW;Canyonleigh | Spring | No | 1/11/17 | Other minimal uses | Populated rangelands | Temperate | Native forests and woodlands |
| 171104SA1 | -35.248 | 138.798 | SA;Paris Creek | Spring | No | 4/11/17 | Modified pastures | Residential rainfed croplands | Temperate | Native forests and woodlands |
| 171104SA3 | -35.248 | 138.798 | SA;Paris Creek | Spring | No | 4/11/17 | Modified pastures | Residential rainfed croplands | Temperate | Native forests and woodlands |
| 171105SA1 | -33.987 | 138.613 | SA;Watervale | Spring | No | 5/11/17 | Cropping | Populated croplands | Temperate | Annual crops and highly modified pastures |
| 171108NSW1 | -34.618 | 149.707 | NSW;Middle Arm | Spring | No | 8/11/17 | Modified pastures | Populated rangelands | Temperate | Native forests and woodlands |
| 171109NSW1 | -34.616 | 149.709 | NSW;Middle Arm | Spring | No | 9/11/17 | Modified pastures | Populated rangelands | Temperate | Native forests and woodlands |
| 171111VIC1 | -38.469 | 144.897 | VIC;Cape Schanck | Spring | No | 11/11/17 | Urban intensive uses | Populated rangelands | Temperate |  |
| 171111VIC2 | -38.470 | 144.897 | VIC;Cape Schanck | Spring | No | 11/11/17 | Urban intensive uses | Populated rangelands | Temperate |  |
| 171119SA1 | -35.648 | 138.187 | SA;Deep Creek | Spring | No | 19/11/17 | Nature conservation | Populated woodlands | Temperate | Native forests and woodlands |
| 171119SA2 | -35.647 | 138.180 | SA;Deep Creek | Spring | No | 19/11/17 | Nature conservation | Populated woodlands | Temperate | Native forests and woodlands |
| 171201SA1 | -33.289 | 136.736 | SA;Yalanda | Summer | No | 1/12/17 | Other minimal uses | Remote croplands | Grassland | Native shrublands and heathlands |
| 171201SA2 | -33.289 | 136.728 | SA;Yalanda | Summer | No | 1/12/17 | Cropping | Remote croplands | Grassland | Annual crops and highly modified pastures |
| 171212SA1 | -34.032 | 140.757 | SA;Uia Riverland | Summer | No | 12/12/17 | Water | Remote rangelands | Grassland | Native forests and woodlands |
| 171226VIC1 | -37.716 | 145.304 | VIC;Christmas Hills | Summer | No | 26/12/17 | Modified pastures | Residential rangelands | Temperate | Annual crops and highly modified pastures |
| 171227WA1 | -31.906 | 116.688 | WA;St Ronans | Summer | No | 27/12/17 | Cropping | Remote croplands | Temperate | Annual crops and highly modified pastures |
| 171231VIC1 | -37.674 | 145.187 | VIC; Neerim Rise | Summer | No | 31/12/17 | Plantations | Urban | Temperate | Native grasslands and minimally modified pastures |
| 180118SA1 | -35.566 | 138.533 | SA;Waitpinga | Summer | No | 8/1/18 | Modified pastures | Populated croplands | Temperate | Annual crops and highly modified pastures |
| 180119SA1 | -35.555 | 139.249 | SA;Narrung | Summer | No | 19/1/18 | Modified pastures | Remote croplands | Grassland | Ephemeral and Permanent Water Features |
| 180204VIC1 | -38.887 | 145.951 | VIC;Tarwin Lower | Summer | No | 4/2/18 | Modified pastures | Populated rangelands | Temperate | Annual crops and highly modified pastures |
| 180215QLD1 | -26.260 | 152.792 | QLD;Mothar Mountain | Summer | No | 16/2/18 | Other minimal uses | Residential woodlands | Subtropical | Native forests and woodlands |
| 180225SA1* | -35.348 | 138.792 | SA;Finnis | Summer | No | 25/2/18 | Modified pastures | Populated croplands | Temperate | Native forests and woodlands |
| 180225SA2* | -35.348 | 138.792 | SA;Finnis | Summer | No | 25/2/18 | Modified pastures | Populated croplands | Temperate | Native forests and woodlands |
| 180303NSW1 | -34.449 | 149.499 | NSW;Crookwell | Autumn | No | 3/3/18 | Modified pastures | Residential rainfed croplands | Temperate | Annual crops and highly modified pastures |
| 180309VIC1 | -38.597 | 143.182 | VIC;Cooriemungle | Autumn | No | 9/3/18 | Grazing of native pastures | Remote croplands | Temperate | Native grasslands and minimally modified pastures |
| 180310SA1 | -37.052 | 140.803 | SA;Struan | Autumn | No | 10/3/18 | Urban intensive uses | Remote croplands | Temperate | Annual crops and highly modified pastures |
| 180313SA1 | -35.011 | 138.629 | SA;Glenalta | Autumn | No | 13/3/18 | Urban intensive uses | Urban | Temperate | Native shrublands and heathlands |
| 180313SA3 | -35.011 | 138.629 | SA;Glenalta | Autumn | No | 13/3/18 | Urban intensive uses | Urban | Temperate | Native shrublands and heathlands |
| 180323SA1 | -35.441 | 138.387 | SA;Wattle Flat | Autumn | No | 23/3/18 | Modified pastures | Populated croplands | Temperate |  |
| 180330SA1 | -35.565 | 139.209 | SA;Narrung | Autumn | No | 30/3/18 | Modified pastures | Remote croplands | Temperate | Annual crops and highly modified pastures |
| 180331NSW2 | -34.750 | 150.496 | NSW;Kangaroo Valley | Autumn | No | 31/3/18 | Other minimal uses | Remote woodlands | Temperate | Native forests and woodlands |
| 180404SA1 | -34.510 | 139.359 | SA;Sedan | Autumn | No | 4/4/18 | Cropping | Remote croplands | Grassland | Annual crops and highly modified pastures |
| 180407SA1* | -35.351 | 138.793 | SA;Finnis | Autumn | No | 7/4/18 | Other minimal uses | Populated croplands | Temperate | Annual crops and highly modified pastures |
| 180407VIC1 | -37.103 | 144.219 | VIC;Campbells Creek | Autumn | No | 7/4/18 | Other minimal uses | Residential rainfed croplands | Temperate | Native forests and woodlands |
| 180411VIC1 | -38.312 | 144.078 | VIC;Gherang | Autumn | No | 11/4/18 | Modified pastures | Populated croplands | Temperate | Native forests and woodlands |
| 180411VIC2 | -38.312 | 144.078 | VIC;Gherang | Autumn | No | 11/4/18 | Other minimal uses | Populated croplands | Temperate | Native forests and woodlands |
| 180412VIC1 | -38.329 | 141.552 | VIC;Portland West | Autumn | No | 12/4/18 | Modified pastures | Remote croplands | Temperate | Annual crops and highly modified pastures |
| 180417QLD1 | -22.110 | 145.195 | QLD;Upper Cornish Creek | Autumn | No | 17/4/18 | Nature conservation | Remote rangelands | Grassland | Native shrublands and heathlands |
| 180418NSW1 | -33.331 | 151.270 | NSW;Somersby | Autumn | No | 18/4/18 | Modified pastures | Populated woodlands | Temperate | Native shrublands and heathlands |
| 180419SA1* | -35.352 | 138.794 | SA;Finnis | Autumn | No | 19/4/18 | Other minimal uses | Populated croplands | Temperate | Annual crops and highly modified pastures |
| 180420SA1* | -35.351 | 138.794 | SA;Finnis | Autumn | No | 20/4/18 | Other minimal uses | Populated croplands | Temperate | Annual crops and highly modified pastures |
| 180422SA1 | -30.448 | 139.031 | SA;Gammon Ranges | Autumn | No | 22/4/18 | Nature conservation | Remote rangelands | Grassland | Native forests and woodlands |
| 180503QLD1 | -15.471 | 145.259 | QLD;Cooktown | Autumn | No | 3/5/18 | Urban intensive uses |  | Tropical |  |
| 180505SA1 | -33.982 | 138.620 | SA;Watervale | Autumn | No | 5/5/18 | Modified pastures | Populated croplands | Temperate | Native forests and woodlands |
| 180520NSW1 | -35.221 | 149.344 | NSW;Wamboin | Autumn | No | 20/5/18 | Rural residential | Populated rangelands | Temperate | Annual crops and highly modified pastures |
| 180530SA1 | -34.597 | 139.342 | SA;Sedan | Autumn | No | 30/5/18 | Modified pastures | Populated croplands | Grassland | Native forests and woodlands |
| 180612SA1 | -30.138 | 136.898 | SA;Arid Recovery | Winter | Yes | 12/6/18 | Grazing of native pastures | Remote rangelands | Desert | Native grasslands and minimally modified pastures |
| 180620SA3 | -35.096 | 139.076 | SA;Monarto | Winter | Yes | 20/6/18 | Nature conservation | Populated croplands | Grassland | Annual crops and highly modified pastures |
| 180621SA1* | -35.350 | 138.789 | SA;Finnis | Winter | Yes | 21/6/18 | Modified pastures | Populated croplands | Temperate | Native forests and woodlands |
| 180623SA3 | -34.545 | 135.918 | SA;Louth Bay | Winter | Yes | 23/6/18 | Mining and waste | Populated croplands | Temperate | Native grasslands and minimally modified pastures |
| 180627SA1 | -30.134 | 139.399 | SA;Arkaroola | Winter | Yes | 27/6/18 | Grazing of native pastures | Remote rangelands | Grassland | Native forests and woodlands |
| 180627SA2 | -30.137 | 139.394 | SA;Arkaroola | Winter | Yes | 27/6/18 | Grazing of native pastures | Remote rangelands | Grassland | Native forests and woodlands |
| 180627SA3 | -30.134 | 139.387 | SA;Arkaroola | Winter | Yes | 27/6/18 | Grazing of native pastures | Remote rangelands | Grassland | Native forests and woodlands |
| 180627SA4 | -30.132 | 139.396 | SA;Arkaroola | Winter | Yes | 27/6/18 | Grazing of native pastures | Remote rangelands | Grassland | Native shrublands and heathlands |
| 180627SA5 | -30.133 | 139.396 | SA;Arkaroola | Winter | Yes | 27/6/18 | Grazing of native pastures | Remote rangelands | Grassland | Native shrublands and heathlands |
| 180627SA6 | -30.133 | 139.397 | SA;Arkaroola | Winter | Yes | 27/6/18 | Grazing of native pastures | Remote rangelands | Grassland | Native forests and woodlands |
| 180627SA7 | -30.134 | 139.387 | SA;Arkaroola | Winter | Yes | 27/6/18 | Grazing of native pastures | Remote rangelands | Grassland | Native forests and woodlands |
| 180628SA1 | -30.135 | 139.397 | SA;Arkaroola | Winter | Yes | 28/6/18 | Grazing of native pastures | Remote rangelands | Grassland | Native forests and woodlands |
| 180628SA2 | -30.139 | 139.395 | SA;Arkaroola | Winter | Yes | 28/6/18 | Grazing of native pastures | Remote rangelands | Grassland | Native forests and woodlands |
| 180628SA3 | -30.140 | 139.395 | SA;Arkaroola | Winter | Yes | 28/6/18 | Grazing of native pastures | Remote rangelands | Grassland | Native forests and woodlands |
| 180628SA4 | -30.141 | 139.396 | SA;Arkaroola | Winter | Yes | 28/6/18 | Grazing of native pastures | Remote rangelands | Grassland | Native forests and woodlands |
| 180628SA5 | -30.145 | 139.398 | SA;Arkaroola | Winter | Yes | 28/6/18 | Grazing of native pastures | Remote rangelands | Grassland | Native grasslands and minimally modified pastures |
| 180628SA6 | -30.145 | 139.398 | SA;Arkaroola | Winter | Yes | 28/6/18 | Grazing of native pastures | Remote rangelands | Grassland | Native grasslands and minimally modified pastures |
| 180628SA7 | -30.163 | 139.426 | SA;Arkaroola | Winter | Yes | 28/6/18 | Grazing of native pastures | Remote rangelands | Grassland | Native grasslands and minimally modified pastures |
| 180628SA8 | -30.163 | 139.427 | SA;Arkaroola | Winter | Yes | 28/6/18 | Grazing of native pastures | Remote rangelands | Grassland | Native grasslands and minimally modified pastures |
| 180628SA9 | -30.162 | 139.437 | SA;Arkaroola | Winter | Yes | 28/6/18 | Grazing of native pastures | Remote rangelands | Grassland | Native forests and woodlands |
| 180701QLD1 | -27.564 | 152.007 | QLD;Withcott | Winter | Yes | 1/7/18 | Rural residential | Populated rangelands | Temperate | Native grasslands and minimally modified pastures |
| 180711WA1 | -16.423 | 125.046 | WA;King Leopold Ranges | Winter | Yes | 9/7/18 | Grazing of native pastures | Remote rangelands | Tropical | Native shrublands and heathlands |
| 180712SA2* | -35.347 | 138.791 | SA;Finnis | Winter | Yes | 12/7/18 | Modified pastures | Populated croplands | Temperate | Native forests and woodlands |
| 180715SA1 | -26.462 | 132.029 | SA;Umuwa | Winter | Yes | 15/7/18 | Managed resource protected areas | Remote rangelands | Grassland | Native grasslands and minimally modified pastures |
| 180716SA1 | -26.462 | 132.029 | SA;Umuwa | Winter | Yes | 16/7/18 | Managed resource protected areas | Remote rangelands | Grassland | Native grasslands and minimally modified pastures |
| 180716SA2 | -26.462 | 132.029 | SA;Umuwa | Winter | Yes | 16/7/18 | Managed resource protected areas | Remote rangelands | Grassland | Native grasslands and minimally modified pastures |
| 180718SA1 | -26.462 | 132.029 | SA;Umuwa | Winter | Yes | 18/7/18 | Managed resource protected areas | Remote rangelands | Grassland | Native grasslands and minimally modified pastures |
| 180718SA2 | -26.462 | 132.029 | SA;Umuwa | Winter | Yes | 18/7/18 | Managed resource protected areas | Remote rangelands | Grassland | Native grasslands and minimally modified pastures |
| 180719SA2 | -26.462 | 132.029 | SA;Umuwa | Winter | Yes | 19/7/18 | Managed resource protected areas | Remote rangelands | Grassland | Native grasslands and minimally modified pastures |
| 180721SA1 | -34.973 | 138.641 | SA;Urrbrae | Winter | Yes | 21/7/18 | Nature conservation | Urban | Temperate | Native forests and woodlands |
| 180725SA1 | -30.373 | 136.844 | SA;Arid Recovery | Winter | Yes | 25/7/18 | Nature conservation | Remote rangelands | Desert | Native grasslands and minimally modified pastures |
| 180726QLD1 | -25.083 | 152.547 | QLD;Woodgate | Winter | Yes | 26/7/18 | Grazing of native pastures | Inhabited treeless and barren lands | Subtropical |  |
| 180729SA1 | -30.373 | 136.844 | SA;Arid Recovery | Winter | Yes | 29/7/18 | Nature conservation | Remote rangelands | Desert | Native grasslands and minimally modified pastures |
| 180729SA2 | -30.373 | 136.844 | SA;Arid Recovery | Winter | Yes | 29/7/18 | Nature conservation | Remote rangelands | Desert | Native grasslands and minimally modified pastures |
| 180729SA3 | -34.964 | 138.648 | SA;Glen Osmond | Winter | Yes | 29/7/18 | Mining and waste | Urban | Temperate | Native forests and woodlands |
| 180729VIC1 | -37.280 | 144.289 | VIC;Denver | Winter | Yes | 29/7/18 | Other minimal uses | Remote rangelands | Temperate | Native forests and woodlands |
| 180730NSW1 | -28.810 | 149.869 | NSW;Garah | Winter | Yes | 30/7/18 | Grazing of native pastures | Remote rangelands | Subtropical | Native forests and woodlands |
| 180809SA1 | -35.265 | 138.870 | SA;Strathalbyn | Winter | Yes | 9/8/18 | Nature conservation | Residential rainfed croplands | Temperate | Annual crops and highly modified pastures |
| 180812NSW1 | -28.688 | 153.581 | NSW;Cooper's Shoot | Winter | Yes | 12/8/18 | Rural residential | Residential woodlands | Subtropical | Native grasslands and minimally modified pastures |
| 180813SA1* | -35.349 | 138.790 | SA;Finnis | Winter | Yes | 13/8/18 | Modified pastures | Populated croplands | Temperate | Native forests and woodlands |
| 180813SA2* | -35.349 | 138.790 | SA;Finnis | Winter | Yes | 13/8/18 | Modified pastures | Populated croplands | Temperate | Native forests and woodlands |
| 180824VIC1 | -36.112 | 146.816 | VIC;West Wodonga | Winter | Yes | 24/8/18 | Modified pastures | Populated croplands | Temperate | Annual crops and highly modified pastures |
| 180831VIC1 | -36.723 | 141.950 | VIC;Natimuk | Winter | Yes | 18/8/31 | Cropping | Residential rainfed croplands | Temperate | Native forests and woodlands |
| 180903SA1 | -34.899 | 138.773 | SA;Montacute | Spring | Yes | 3/9/18 | Irrigated horticulture | Residential woodlands | Temperate | Horticultural trees and shrubs |
| 180911SA1 | -34.117 | 140.808 | SA;Murtho | Spring | Yes | 11/9/18 | Irrigated horticulture | Populated rangelands | Grassland | Annual crops and highly modified pastures |
| 180912SA1 | -34.901 | 138.872 | SA;Lobethal | Spring | Yes | 12/9/18 | Modified pastures | Residential woodlands | Temperate | Annual crops and highly modified pastures |
| 180915SA1 | -35.160 | 138.559 | SA;Onkaparinga | Spring | Yes | 15/9/18 | Nature conservation | Urban | Temperate | Horticultural trees and shrubs |
| 180925SA1* | -35.739 | 137.636 | SA;Kangaroo Island | Spring | Yes | 25/9/18 | Modified pastures | Populated croplands | Temperate |  |
| 180927SA1 | -30.373 | 136.844 | SA;Arid Recovery | Spring | Yes | 27/9/18 | Nature conservation | Remote rangelands | Desert | Native grasslands and minimally modified pastures |
| 180930NSW1 | -30.517 | 151.738 | NSW;Armidale | Spring | Yes | 30/9/18 | Modified pastures | Mixed settlements | Temperate | Annual crops and highly modified pastures |
| 181012VIC1 | -37.646 | 149.700 | VIC;Wingan River | Spring | No | 12/10/18 | Nature conservation | Remote woodlands | Temperate | Native forests and woodlands |
| 181016NSW1 | -36.440 | 148.556 | NSW;Crackenback | Spring | No | 16/10/18 | Other minimal uses | Populated rangelands | Temperate | Native forests and woodlands |
| 181016NSW2 | -34.279 | 146.045 | NSW;Griffith | Spring | No | 16/10/18 | Urban intensive uses | Mixed settlements | Grassland | Built-up |
| 181018SA1 | -30.373 | 136.844 | SA;Arid Recovery | Spring | No | 8/10/18 | Nature conservation | Remote rangelands | Desert | Native grasslands and minimally modified pastures |
| 181025SA1 | -33.289 | 138.262 | SA;Huddlestone | Spring | No | 25/10/18 | Other minimal uses | Remote croplands | Temperate | Annual crops and highly modified pastures |
| 181026SA1 | -33.289 | 138.261 | SA;Huddlestone | Spring | No | 26/10/18 | Other minimal uses | Remote croplands | Temperate | Annual crops and highly modified pastures |
| 181027SA1 | -33.287 | 138.262 | SA;Huddlestone | Spring | No | 27/10/18 | Other minimal uses | Remote croplands | Temperate | Annual crops and highly modified pastures |
| 181027SA2 | -33.287 | 138.261 | SA;Huddlestone | Spring | No | 27/10/18 | Other minimal uses | Remote croplands | Temperate | Annual crops and highly modified pastures |
| 181028SA1 | -33.289 | 138.260 | SA;Huddlestone | Spring | No | 28/10/18 | Other minimal uses | Remote croplands | Temperate | Annual crops and highly modified pastures |
| 181028SA2 | -33.289 | 138.262 | SA;Huddlestone | Spring | No | 28/10/18 | Cropping | Remote croplands | Temperate | Native forests and woodlands |
| 181107QLD1 | -26.319 | 148.757 | QLD;Eumamurrin | Spring | No | 7/11/18 | Cropping | Remote rangelands | Subtropical | Native grasslands and minimally modified pastures |
| 181107QLD2 | -26.319 | 148.757 | QLD;Eumamurrin | Spring | No | 7/11/18 | Cropping | Remote rangelands | Subtropical | Native grasslands and minimally modified pastures |
| 181115SA2 | -35.114 | 139.265 | SA;Murray Bridge | Spring | No | 15/11/18 | Urban intensive uses | Mixed settlements | Grassland | Native forests and woodlands |
| 181125SA1 | -35.567 | 138.482 | SA;Waitpinga | Spring | No | 25/11/18 | Cropping | Populated croplands | Temperate | Annual crops and highly modified pastures |
| 181231VIC1 | -38.344 | 146.769 | VIC;Willung South | Summer | No | 31/12/18 | Modified pastures | Wild woodlands | Temperate | Annual crops and highly modified pastures |
| 190103QLD1 | -27.462 | 152.935 | QLD;The Gap | Summer | No | 3/1/19 | Rural residential | Urban | Subtropical | Native forests and woodlands |
| 190126VIC1 | -38.330 | 146.759 | VIC;Willung South | Summer | No | 26/1/19 | Modified pastures | Wild woodlands | Temperate | Native grasslands and minimally modified pastures |
| 190312ACT1 | -35.354 | 149.085 | ACT;Chifley | Autumn | No | 12/3/19 | Urban intensive uses | Urban | Temperate | Built-up |
| 190402VIC1 | -36.846 | 144.469 | VIC;Eppalock | Autumn | No | 2/4/19 | Nature conservation | Populated croplands | Temperate | Native forests and woodlands |
| 190402VIC2 | -36.846 | 144.471 | VIC;Eppalock | Autumn | No | 2/4/19 | Nature conservation | Populated croplands | Temperate | Native forests and woodlands |
| 190424SA1* | -35.350 | 138.791 | SA;Finnis | Autumn | No | 24/4/19 | Modified pastures | Populated croplands | Temperate | Native forests and woodlands |
| 190424SA2* | -35.350 | 138.791 | SA;Finnis | Autumn | No | 24/4/19 | Modified pastures | Populated croplands | Temperate | Native forests and woodlands |
| 190424SA3* | -35.350 | 138.791 | SA;Finnis | Autumn | No | 24/4/19 | Modified pastures | Populated croplands | Temperate | Native forests and woodlands |
| 190427SA1 | -35.555 | 139.249 | SA;Narrung | Autumn | No | 27/4/19 | Modified pastures | Remote croplands | Grassland | Ephemeral and Permanent Water Features |
| 190430NSW1 | -32.378 | 149.702 | NSW;Cook's Gap | Autumn | No | 30/4/19 | Rural residential | Remote rangelands | Temperate | Native forests and woodlands |
| 190430NSW2 | -29.250 | 150.751 | NSW;Coolatai | Autumn | No | 30/4/19 | Urban intensive uses | Remote rangelands | Temperate | Native forests and woodlands |
| 190502NSW1 | -29.021 | 151.935 | NSW;Tenterfield | Autumn | No | 2/5/19 | Modified pastures | Remote rangelands | Temperate | Native forests and woodlands |
| 190502NSW2 | -29.021 | 151.935 | NSW;Tenterfield | Autumn | No | 2/5/19 | Modified pastures | Remote rangelands | Temperate | Native forests and woodlands |
| 190502NSW3 | -29.021 | 151.935 | NSW;Tenterfield | Autumn | No | 2/5/19 | Modified pastures | Remote rangelands | Temperate | Native forests and woodlands |
| 190508SA1 | -31.272 | 138.388 | SA;Parachilna | Autumn | No | 8/5/19 | Grazing of native pastures | Remote rangelands | Desert | Native shrublands and heathlands |
| 190508SA2 | -31.275 | 138.375 | SA;Parachilna | Autumn | No | 8/5/19 | Grazing of native pastures | Remote rangelands | Desert | Native shrublands and heathlands |
| 190508SA3 | -31.275 | 138.375 | SA;Parachilna | Autumn | No | 8/5/19 | Grazing of native pastures | Remote rangelands | Desert | Native shrublands and heathlands |
| 190523QLD1 | -27.846 | 150.155 | QLD;Southwood | Autumn | No | 23/5/19 | Grazing of native pastures | Remote rangelands | Subtropical | Native forests and woodlands |
| 190601NSW1 | -29.237 | 152.013 | NSW;Sandy Flat | Winter | Yes | 1/6/19 | Modified pastures | Remote rangelands | Temperate | Annual crops and highly modified pastures |
| 190603SA1 | -31.274 | 138.381 | SA;Parachilna | Winter | Yes | 3/6/19 | Grazing of native pastures | Remote rangelands | Desert | Native shrublands and heathlands |
| 190603SA3 | -31.274 | 138.381 | SA;Parachilna | Winter | Yes | 3/6/19 | Grazing of native pastures | Remote rangelands | Desert | Native shrublands and heathlands |
| 190610SA1 | -35.256 | 138.728 | SA;McHarg Creek | Winter | Yes | 10/6/19 | Other minimal uses | Populated woodlands | Temperate | Annual crops and highly modified pastures |
| 190727WA2 | -26.515 | 114.098 | WA;Hamelin Pool | Winter | Yes | 27/7/19 | Grazing of native pastures | Remote rangelands | Grassland | Native shrublands and heathlands |
| 190801WA1 | -27.525 | 114.529 | WA;Eurardy | Winter | Yes | 1/8/19 | Grazing of native pastures | Remote rangelands | Grassland | Native shrublands and heathlands |
| 190801WA2 | -27.605 | 114.693 | WA;Eurardy | Winter | Yes | 1/8/19 | Grazing of native pastures | Remote rangelands | Grassland | Native shrublands and heathlands |
| 190812NSW1 | -33.583 | 149.263 | NSW;Blayney | Winter | Yes | 12/8/19 | Modified pastures | Residential rainfed croplands | Temperate | Native forests and woodlands |
| 190812NSW2 | -33.583 | 149.263 | NSW;Blayney | Winter | Yes | 12/8/19 | Modified pastures | Residential rainfed croplands | Temperate | Native forests and woodlands |
| 190813WA1 | -16.081 | 124.469 | WA;Derby West Kimberley | Winter | Yes | 13/8/19 | Other minimal uses |  | Tropical | Native grasslands and minimally modified pastures |
| 190817SA1 | -34.947 | 138.691 | SA;Greenhill | Winter | Yes | 17/8/19 | Other minimal uses | Mixed settlements | Temperate | Built-up |
| 190822WA1 | -16.688 | 125.246 | WA;King Leopold Ranges | Winter | Yes | 22/8/19 | Grazing of native pastures | Remote rangelands | Tropical | Native shrublands and heathlands |
| 190903NSW1 | -34.847 | 149.085 | NSW;Lade Vale | Spring | Yes | 3/9/19 | Modified pastures | Remote croplands | Temperate | Annual crops and highly modified pastures |
| 190903TAS1 | -43.097 | 147.964 | TAS;Fortescue | Spring | Yes | 3/9/19 | Nature conservation | Inhabited treeless and barren lands | Temperate |  |
| 190930QLD1 | -27.317 | 152.796 | QLD;Cedar Creek | Spring | Yes | 30/9/19 | Other minimal uses | Populated woodlands | Subtropical | Native grasslands and minimally modified pastures |

*Scat collected within one day of depositing from citizen scientist’s property **Table S2: Information for faecal samples collected in captivity.** UMD = Updated Meat Diet; DOC = Date of Collection.

| Sample ID | Echidna Name | Diet | Sex | Zoo | Season | Breeding Season | DOC |
| --- | --- | --- | --- | --- | --- | --- | --- |
| BL231018 | Blue | Meat | Female | Perth | Spring | No | 23/10/18 |
| BL241018 | Blue | Meat | Female | Perth | Spring | No | 24/10/18 |
| BL251018 | Blue | Meat | Female | Perth | Spring | No | 25/10/18 |
| CH240818 | Chindi | Meat | Female | Perth | Winter | Yes | 24/8/18 |
| CH250818 | Chindi | Meat | Female | Perth | Winter | Yes | 25/8/18 |
| CH260818 | Chindi | Meat | Female | Perth | Winter | Yes | 26/8/18 |
| CO230218 | Cojine | Meat | Male | Perth | Summer | No | 23/2/18 |
| CO2302181^a^ | Cojine | Meat | Male | Perth | Summer | No | 23/2/18 |
| CO2302182^a^ | Cojine | Meat | Male | Perth | Summer | No | 23/2/18 |
| CO2302183^a^ | Cojine | Meat | Male | Perth | Summer | No | 23/2/18 |
| CO240218 | Cojine | Meat | Male | Perth | Summer | No | 24/2/18 |
| CO250218 | Cojine | Meat | Male | Perth | Summer | No | 25/2/18 |
| CO2502181^a^ | Cojine | Meat | Male | Perth | Summer | No | 25/2/18 |
| CO2502182^a^ | Cojine | Meat | Male | Perth | Summer | No | 25/2/18 |
| CO2502183^a^ | Cojine | Meat | Male | Perth | Summer | No | 25/2/18 |
| GR231018 | Green | Meat | Female | Perth | Spring | No | 23/10/18 |
| GR241018 | Green | Meat | Female | Perth | Spring | No | 24/10/18 |
| GR251018 | Green | Meat | Female | Perth | Spring | No | 25/10/18 |
| JI240718 | Jilba | Meat | Female | Perth | Winter | Yes | 24/7/18 |
| JI250718 | Jilba | Meat | Female | Perth | Winter | Yes | 25/7/18 |
| JI260718 | Jilba | Meat | Female | Perth | Winter | Yes | 26/7/18 |
| KA240718 | Kain | Meat | Male | Perth | Winter | Yes | 24/7/18 |
| KA250718 | Kain | Meat | Male | Perth | Winter | Yes | 25/7/18 |
| KA260718 | Kain | Meat | Male | Perth | Winter | Yes | 26/7/18 |
| MI241018 | Mila | Meat | Female | Perth | Spring | No | 24/10/18 |
| MI251018 | Mila | Meat | Female | Perth | Spring | No | 25/10/18 |
| MI261018 | Mila | Meat | Female | Perth | Spring | No | 26/10/18 |
| MO230718 | Moa | Meat | Female | Perth | Winter | Yes | 23/7/18 |
| MO240718 | Moa | Meat | Female | Perth | Winter | Yes | 24/7/18 |
| MO250718 | Moa | Meat | Female | Perth | Winter | Yes | 25/7/18 |
| NY260218 | Nyingarn | Meat | Male | Perth | Summer | No | 26/2/18 |
| NY270218 | Nyingarn | Meat | Male | Perth | Summer | No | 27/2/18 |
| MSSBE175 | Snorky | UMD | Female | Taronga | Spring | No | 17/10/19 |
| MSSBE181 | Rose | UMD | Female | Taronga | Spring | No | 17/10/19 |
| MSSBE193 | Bristle | UMD | Male | Taronga | Autumn | No | 3/5/19 |
| MSSBE197 | Bali | UMD | Female | Taronga | Winter | Yes | 7/8/19 |
| MSSBE202 | Rex | UMD | Male | Taronga | Spring | Yes | 18/9/19 |
| MSSBE111 | Bristle | Vetafarm | Male | Taronga | Spring | No | 21/11/19 |
| MSSBE140 | Leroy | Vetafarm | Female | Taronga | Summer | No | 13/12/18 |
| MSSBE187 | Bristle | Vetafarm | Male | Taronga | Autumn | No | 24/4/19 |
| MSSBE210 | Bali | Vetafarm | Female | Taronga | Winter | Yes | 19/7/19 |
| MSSBE61 | Spike | Vetafarm | Female | Taronga | Summer | No | 6/2/17 |
| MSSBE62 | Spike | Vetafarm | Female | Taronga | Summer | No | 7/2/17 |
| MSSBE63 | Spike | Vetafarm | Female | Taronga | Summer | No | 11/2/17 |
| MSSBE205 | Bali | Wombaroo | Female | Taronga | Spring | Yes | 18/9/19 |
| MSSBE224 | Ganyi | Wombaroo | Female | Taronga | Summer | No | 16/12/19 |
| MSSBE225 | Ganyi | Wombaroo | Female | Taronga | Summer | No | 17/12/19 |
| MSSBE226 | Ganyi | Wombaroo | Female | Taronga | Summer | No | 18/12/19 |
| MSSBE235 | Jindi | Wombaroo | Male | Taronga | Summer | No | 5/12/19 |
| MSSBE236 | Gemma | Wombaroo | Female | Taronga | Summer | No | 9/12/19 |

^a^Technical triplicates, same day extraction; not included in statistical analyses but to evaluate extraction technique (see Figure S6)

**Table S3: Ingredients for the four different diets fed to echidnas in captivity.**

| Ingredients | | | |
| --- | --- | --- | --- |
| Perth Zoo’s Meat Diet | **Taronga Zoo’s Updated Meat Diet** | **Taronga Zoo’s Vetafarm Diet** | **Taronga Zoo’s Wombaroo Diet** |
| Beef, lean fine mince | Beef, lean fine mince | Meat Meal | Meat Meal |
| Microcrystalline cellulose | Microcrystalline cellulose | Corn | Soy and protein isolates, cellulose |
| Egg, hardboiled | Egg, hardboiled | Roughage (straw) | Processed cereals (wheat and rice) |
| Banana | KER bone food | Potato starch | Vegetable oil, Omega 3 & 6 fatty acids |
| Pentavite with iron multivitamin | Pentavite with iron multivitamin | Minerals (calcium, chloride, cobalt, copper, iodine, iron, magnesium, manganese, phosphorous, potassium, sodium, sulphur, selenium and zinc) | Minerals (calcium, chloride, cobalt, copper, iodine, iron, magnesium, manganese, phosphorous, potassium, sodium, sulphur, selenium and zinc) |
| Calcium carbonate | Calcium carbonate | Vegetable oils and organic acids | Amino acids (lysine, methionine, taurine) |
| Mealworms | KER nano E | Amino acids (arginine, cysteine, histidine, isoleucine, leucine, lysine, methionine, phenylalanine, taurine, threonine, tryptophan, tyrosine & valine) | Vitamins: A, B1, B2, B3, B5, B6, B9, B12, C, D3, K, biotin & choline |
|  |  |  | Natuzyme multi-enzyme Actigen® dried yeast prebiotic |

**Table S4: Wild echidna alpha diversity statistics.** Green rows = statistically significant; PD = Phylogenetic Diversity; ASVs = Amplicon Sequence Variants.

|  | **Group 1** | **Group 2** | **H** | **p-value** | **q-value** |
| --- | --- | --- | --- | --- | --- |
| **Faith's PD** | Desert (n=11) | Grassland (n=39) | 2.0075 | 0.1565 | 0.2236 |
|  | Desert (n=11) | Subtropical (n=9) | 5.7273 | 0.0167 | 0.0939 |
|  | Desert (n=11) | Temperate (n=96) | 0.6567 | 0.4177 | 0.4893 |
|  | Desert (n=11) | Tropical (n=4) | 5.5227 | 0.0188 | 0.0939 |
|  | Grassland (n=39) | Subtropical (n=9) | 4.0832 | 0.0433 | 0.1444 |
|  | Grassland (n=39) | Temperate (n=96) | 0.1743 | 0.6763 | 0.6763 |
|  | Grassland (n=39) | Tropical (n=4) | 2.0210 | 0.1551 | 0.2236 |
|  | Subtropical (n=9) | Temperate (n=96) | 3.3543 | 0.0670 | 0.1676 |
|  | Subtropical (n=9) | Tropical (n=4) | 0.5952 | 0.4404 | 0.4893 |
|  | Temperate (n=96) | Tropical (n=4) | 2.8515 | 0.0913 | 0.1826 |
| **Observed ASVs** | Desert (n=11) | Grassland (n=39) | 2.0413 | 0.1531 | 0.2356 |
|  | Desert (n=11) | Subtropical (n=9) | 5.3694 | 0.0205 | 0.1025 |
|  | Desert (n=11) | Temperate (n=96) | 0.7783 | 0.3777 | 0.4196 |
|  | Desert (n=11) | Tropical (n=4) | 5.5227 | 0.0188 | 0.1025 |
|  | Grassland (n=39) | Subtropical (n=9) | 3.5671 | 0.0589 | 0.1179 |
|  | Grassland (n=39) | Temperate (n=96) | 0.5846 | 0.4445 | 0.4445 |
|  | Grassland (n=39) | Tropical (n=4) | 1.1370 | 0.2863 | 0.3579 |
|  | Subtropical (n=9) | Temperate (n=96) | 4.3167 | 0.0377 | 0.1179 |
|  | Subtropical (n=9) | Tropical (n=4) | 1.9286 | 0.1649 | 0.2356 |
|  | Temperate (n=96) | Tropical (n=4) | 3.6764 | 0.0552 | 0.1179 |
| **Shannon's Diversity** | Desert (n=11) | Grassland (n=39) | 0.2305 | 0.6312 | 0.7013 |
|  | Desert (n=11) | Subtropical (n=9) | 4.0534 | 0.0441 | 0.2016 |
|  | Desert (n=11) | Temperate (n=96) | 0.3788 | 0.5383 | 0.6728 |
|  | Desert (n=11) | Tropical (n=4) | 2.8807 | 0.0896 | 0.2241 |
|  | Grassland (n=39) | Subtropical (n=9) | 4.8646 | 0.0274 | 0.2016 |
|  | Grassland (n=39) | Temperate (n=96) | 0.0323 | 0.8575 | 0.8575 |
|  | Grassland (n=39) | Tropical (n=4) | 1.1818 | 0.2770 | 0.3957 |
|  | Subtropical (n=9) | Temperate (n=96) | 3.5241 | 0.0605 | 0.2016 |
|  | Subtropical (n=9) | Tropical (n=4) | 2.3810 | 0.1228 | 0.2456 |
|  | Temperate (n=96) | Tropical (n=4) | 1.3889 | 0.2386 | 0.3957 |
| **Pileou's Evenness** | Desert (n=11) | Grassland (n=39) | 0.0001 | 0.9907 | 0.9907 |
|  | Desert (n=11) | Subtropical (n=9) | 2.9221 | 0.0874 | 0.3310 |
|  | Desert (n=11) | Temperate (n=96) | 0.0085 | 0.9264 | 0.9907 |
|  | Desert (n=11) | Tropical (n=4) | 0.4261 | 0.5139 | 0.6880 |
|  | Grassland (n=39) | Subtropical (n=9) | 4.2995 | 0.0381 | 0.3310 |
|  | Grassland (n=39) | Temperate (n=96) | 0.3565 | 0.5504 | 0.6880 |
|  | Grassland (n=39) | Tropical (n=4) | 1.2745 | 0.2589 | 0.6161 |
|  | Subtropical (n=9) | Temperate (n=96) | 2.7170 | 0.0993 | 0.3310 |
|  | Subtropical (n=9) | Tropical (n=4) | 0.8571 | 0.3545 | 0.6161 |
|  | Temperate (n=96) | Tropical (n=4) | 0.8048 | 0.3697 | 0.6161 |

**Table S5: Wild echidna beta diversity statistics.** Green rows = statistically significant.

|  | **Group 1** | **Group 2** | **Sample size** | **Permutations** | **pseudo-F** | **p-value** | **q-value** |
| --- | --- | --- | --- | --- | --- | --- | --- |
| **Season** | Autumn | Spring | 89 | 999 | 1.837 | 0.021 | 0.033 |
|  | Autumn | Summer | 54 | 999 | 1.034 | 0.357 | 0.357 |
|  | Autumn | Winter | 94 | 999 | 3.260 | 0.001 | 0.006 |
|  | Spring | Summer | 65 | 999 | 1.344 | 0.106 | 0.127 |
|  | Spring | Winter | 105 | 999 | 1.726 | 0.022 | 0.033 |
|  | Summer | Winter | 70 | 999 | 1.906 | 0.015 | 0.033 |
| **Ecoregions** | Carnavon Xeric Shrubs | Eastern Asutralia Temperate Forests | 45 | 999 | 1.006 | 0.412 | 0.721 |
|  | Carnavon Xeric Shrubs | Great Sandy-Tanami-Central Ranges Desert | 7 | 999 | 1.706 | 0.272 | 0.708 |
|  | Carnavon Xeric Shrubs | Northern Australia and Trans-Fly Savannas | 5 | 999 | 0.879 | 0.794 | 0.924 |
|  | Carnavon Xeric Shrubs | Southern Australia Mallee and Woodlands | 64 | 999 | 0.970 | 0.496 | 0.744 |
|  | Carnavon Xeric Shrubs | Southwestern Australia Forests and Scrub | 4 | 999 | 0.775 | 1.000 | 1.000 |
|  | Eastern Asutralia Temperate Forests | Great Sandy-Tanami-Central Ranges Desert | 50 | 999 | 2.323 | 0.003 | 0.028 |
|  | Eastern Asutralia Temperate Forests | Northern Australia and Trans-Fly Savannas | 48 | 999 | 1.017 | 0.371 | 0.708 |
|  | Eastern Asutralia Temperate Forests | Southern Australia Mallee and Woodlands | 107 | 999 | 1.630 | 0.034 | 0.137 |
|  | Eastern Asutralia Temperate Forests | Southwestern Australia Forests and Scrub | 47 | 999 | 0.767 | 0.794 | 0.924 |
|  | Great Sandy-Tanami-Central Ranges Desert | Northern Australia and Trans-Fly Savannas | 10 | 999 | 1.013 | 0.359 | 0.708 |
|  | Great Sandy-Tanami-Central Ranges Desert | Southern Australia Mallee and Woodlands | 69 | 999 | 2.394 | 0.003 | 0.028 |
|  | Great Sandy-Tanami-Central Ranges Desert | Southwestern Australia Forests and Scrub | 9 | 999 | 1.377 | 0.178 | 0.534 |
|  | Northern Australia and Trans-Fly Savannas | Southern Australia Mallee and Woodlands | 67 | 999 | 1.082 | 0.327 | 0.708 |
|  | Northern Australia and Trans-Fly Savannas | Southwestern Australia Forests and Scrub | 7 | 999 | 0.657 | 1.000 | 1.000 |
|  | Southern Australia Mallee and Woodlands | Southwestern Australia Forests and Scrub | 66 | 999 | 0.762 | 0.836 | 0.924 |
| **Anthropogenic Biomes** | Inhabited treeless and barren lands | Mixed settlements | 7 | 999 | 1.247 | 0.261 | 0.703 |
|  | Inhabited treeless and barren lands | Populated croplands | 37 | 999 | 1.480 | 0.047 | 0.535 |
|  | Inhabited treeless and barren lands | Populated rangelands | 14 | 999 | 1.422 | 0.094 | 0.546 |
|  | Inhabited treeless and barren lands | Populated woodlands | 7 | 999 | 1.510 | 0.196 | 0.703 |
|  | Inhabited treeless and barren lands | Remote croplands | 19 | 999 | 1.832 | 0.035 | 0.535 |
|  | Inhabited treeless and barren lands | Remote rangelands | 55 | 999 | 1.368 | 0.093 | 0.546 |
|  | Inhabited treeless and barren lands | Remote woodlands | 4 | 999 | 1.326 | 0.336 | 0.703 |
|  | Inhabited treeless and barren lands | Residential rainfed croplands | 11 | 999 | 1.753 | 0.032 | 0.535 |
|  | Inhabited treeless and barren lands | Residential rangelands | 3 | 999 | 1.962 | 0.364 | 0.703 |
|  | Inhabited treeless and barren lands | Residential woodlands | 7 | 999 | 1.655 | 0.095 | 0.546 |
|  | Inhabited treeless and barren lands | Urban | 11 | 999 | 1.496 | 0.062 | 0.546 |
|  | Inhabited treeless and barren lands | Wild woodlands | 4 | 999 | 2.535 | 0.336 | 0.703 |
|  | Mixed settlements | Populated croplands | 40 | 999 | 1.035 | 0.371 | 0.703 |
|  | Mixed settlements | Populated rangelands | 17 | 999 | 0.945 | 0.418 | 0.732 |
|  | Mixed settlements | Populated woodlands | 10 | 999 | 0.788 | 0.708 | 0.871 |
|  | Mixed settlements | Remote croplands | 22 | 999 | 0.914 | 0.543 | 0.818 |
|  | Mixed settlements | Remote rangelands | 58 | 999 | 1.021 | 0.397 | 0.723 |
|  | Mixed settlements | Remote woodlands | 7 | 999 | 0.520 | 0.949 | 0.975 |
|  | Mixed settlements | Residential rainfed croplands | 14 | 999 | 1.117 | 0.300 | 0.703 |
|  | Mixed settlements | Residential rangelands | 6 | 999 | 1.066 | 0.534 | 0.818 |
|  | Mixed settlements | Residential woodlands | 10 | 999 | 0.987 | 0.455 | 0.753 |
|  | Mixed settlements | Urban | 14 | 999 | 0.752 | 0.834 | 0.972 |
|  | Mixed settlements | Wild woodlands | 7 | 999 | 0.929 | 0.556 | 0.818 |
|  | Populated croplands | Populated rangelands | 47 | 999 | 1.063 | 0.335 | 0.703 |
|  | Populated croplands | Populated woodlands | 40 | 999 | 1.100 | 0.278 | 0.703 |
|  | Populated croplands | Remote croplands | 52 | 999 | 1.028 | 0.361 | 0.703 |
|  | Populated croplands | Remote rangelands | 88 | 999 | 1.979 | 0.013 | 0.535 |
|  | Populated croplands | Remote woodlands | 37 | 999 | 0.738 | 0.898 | 0.972 |
|  | Populated croplands | Residential rainfed croplands | 44 | 999 | 1.112 | 0.262 | 0.703 |
|  | Populated croplands | Residential rangelands | 36 | 999 | 1.431 | 0.070 | 0.546 |
|  | Populated croplands | Residential woodlands | 40 | 999 | 0.900 | 0.585 | 0.819 |
|  | Populated croplands | Urban | 44 | 999 | 1.059 | 0.326 | 0.703 |
|  | Populated croplands | Wild woodlands | 37 | 999 | 1.260 | 0.139 | 0.633 |
|  | Populated rangelands | Populated woodlands | 17 | 999 | 1.264 | 0.159 | 0.633 |
|  | Populated rangelands | Remote croplands | 29 | 999 | 1.030 | 0.363 | 0.703 |
|  | Populated rangelands | Remote rangelands | 65 | 999 | 1.821 | 0.038 | 0.535 |
|  | Populated rangelands | Remote woodlands | 14 | 999 | 0.602 | 0.891 | 0.972 |
|  | Populated rangelands | Residential rainfed croplands | 21 | 999 | 1.314 | 0.160 | 0.633 |
|  | Populated rangelands | Residential rangelands | 13 | 999 | 1.613 | 0.076 | 0.546 |
|  | Populated rangelands | Residential woodlands | 17 | 999 | 1.005 | 0.402 | 0.723 |
|  | Populated rangelands | Urban | 21 | 999 | 1.116 | 0.268 | 0.703 |
|  | Populated rangelands | Wild woodlands | 14 | 999 | 1.427 | 0.124 | 0.627 |
|  | Populated woodlands | Remote croplands | 22 | 999 | 0.911 | 0.536 | 0.818 |
|  | Populated woodlands | Remote rangelands | 58 | 999 | 1.173 | 0.235 | 0.703 |
|  | Populated woodlands | Remote woodlands | 7 | 999 | 0.591 | 0.950 | 0.975 |
|  | Populated woodlands | Residential rainfed croplands | 14 | 999 | 0.911 | 0.565 | 0.818 |
|  | Populated woodlands | Residential rangelands | 6 | 999 | 0.926 | 0.658 | 0.843 |
|  | Populated woodlands | Residential woodlands | 10 | 999 | 0.794 | 0.757 | 0.915 |
|  | Populated woodlands | Urban | 14 | 999 | 0.782 | 0.764 | 0.915 |
|  | Populated woodlands | Wild woodlands | 7 | 999 | 0.651 | 0.959 | 0.975 |
|  | Remote croplands | Remote rangelands | 70 | 999 | 1.582 | 0.046 | 0.535 |
|  | Remote croplands | Remote woodlands | 19 | 999 | 0.625 | 0.907 | 0.972 |
|  | Remote croplands | Residential rainfed croplands | 26 | 999 | 0.986 | 0.430 | 0.736 |
|  | Remote croplands | Residential rangelands | 18 | 999 | 1.475 | 0.096 | 0.546 |
|  | Remote croplands | Residential woodlands | 22 | 999 | 0.712 | 0.859 | 0.972 |
|  | Remote croplands | Urban | 26 | 999 | 1.058 | 0.345 | 0.703 |
|  | Remote croplands | Wild woodlands | 19 | 999 | 1.054 | 0.405 | 0.723 |
|  | Remote rangelands | Remote woodlands | 55 | 999 | 0.885 | 0.632 | 0.843 |
|  | Remote rangelands | Residential rainfed croplands | 62 | 999 | 1.877 | 0.015 | 0.535 |
|  | Remote rangelands | Residential rangelands | 54 | 999 | 1.370 | 0.045 | 0.535 |
|  | Remote rangelands | Residential woodlands | 58 | 999 | 1.147 | 0.248 | 0.703 |
|  | Remote rangelands | Urban | 62 | 999 | 1.580 | 0.056 | 0.546 |
|  | Remote rangelands | Wild woodlands | 55 | 999 | 1.281 | 0.155 | 0.633 |
|  | Remote woodlands | Residential rainfed croplands | 11 | 999 | 0.666 | 0.964 | 0.975 |
|  | Remote woodlands | Residential rangelands | 3 | 999 | 1.002 | 0.686 | 0.855 |
|  | Remote woodlands | Residential woodlands | 7 | 999 | 0.694 | 0.865 | 0.972 |
|  | Remote woodlands | Urban | 11 | 999 | 0.630 | 0.882 | 0.972 |
|  | Remote woodlands | Wild woodlands | 4 | 999 | 0.711 | 1.000 | 1.000 |
|  | Residential rainfed croplands | Residential rangelands | 10 | 999 | 1.403 | 0.105 | 0.562 |
|  | Residential rainfed croplands | Residential woodlands | 14 | 999 | 0.823 | 0.623 | 0.843 |
|  | Residential rainfed croplands | Urban | 18 | 999 | 1.064 | 0.358 | 0.703 |
|  | Residential rainfed croplands | Wild woodlands | 11 | 999 | 0.993 | 0.321 | 0.703 |
|  | Residential rangelands | Residential woodlands | 6 | 999 | 1.390 | 0.160 | 0.633 |
|  | Residential rangelands | Urban | 10 | 999 | 1.175 | 0.201 | 0.703 |
|  | Residential rangelands | Wild woodlands | 3 | 999 | 1.664 | 0.335 | 0.703 |
|  | Residential woodlands | Urban | 14 | 999 | 0.975 | 0.437 | 0.736 |
|  | Residential woodlands | Wild woodlands | 7 | 999 | 1.127 | 0.218 | 0.703 |
|  | Urban | Wild woodlands | 11 | 999 | 1.011 | 0.486 | 0.790 |
| **Koppen Climate 2** | Desert | Grassland | 50 | 999 | 1.396 | 0.140 | 0.280 |
|  | Desert | Subtropical | 20 | 999 | 1.246 | 0.209 | 0.348 |
|  | Desert | Temperate | 107 | 999 | 1.451 | 0.075 | 0.280 |
|  | Desert | Tropical | 15 | 999 | 0.985 | 0.456 | 0.529 |
|  | Grassland | Subtropical | 48 | 999 | 1.377 | 0.131 | 0.280 |
|  | Grassland | Temperate | 135 | 999 | 3.091 | 0.001 | 0.010 |
|  | Grassland | Tropical | 43 | 999 | 0.925 | 0.476 | 0.529 |
|  | Subtropical | Temperate | 105 | 999 | 1.337 | 0.124 | 0.280 |
|  | Subtropical | Tropical | 13 | 999 | 0.700 | 0.903 | 0.903 |
|  | Temperate | Tropical | 100 | 999 | 1.066 | 0.331 | 0.473 |
| **Koppen Climate 1** | Desert: hot (persistently dry) | Grassland: hot (persistently dry) | 18 | 999 | 1.903 | 0.037 | 0.366 |
|  | Desert: hot (persistently dry) | Grassland: hot (summer drought) | 14 | 999 | 1.025 | 0.360 | 0.609 |
|  | Desert: hot (persistently dry) | Grassland: warm (persistently dry) | 38 | 999 | 1.401 | 0.126 | 0.480 |
|  | Desert: hot (persistently dry) | Subtropical: moderately dry winter | 14 | 999 | 1.238 | 0.165 | 0.503 |
|  | Desert: hot (persistently dry) | Subtropical: no dry season | 16 | 999 | 0.968 | 0.501 | 0.701 |
|  | Desert: hot (persistently dry) | Temperate: distinctly dry (and hot) summer | 18 | 999 | 1.170 | 0.256 | 0.522 |
|  | Desert: hot (persistently dry) | Temperate: distinctly dry (and warm) summer | 60 | 999 | 1.484 | 0.060 | 0.366 |
|  | Desert: hot (persistently dry) | Temperate: no dry season (hot summer) | 15 | 999 | 1.163 | 0.261 | 0.522 |
|  | Desert: hot (persistently dry) | Temperate: no dry season (mild summer) | 15 | 999 | 0.927 | 0.555 | 0.733 |
|  | Desert: hot (persistently dry) | Temperate: no dry season (warm summer) | 46 | 999 | 1.494 | 0.068 | 0.374 |
|  | Desert: hot (persistently dry) | Tropical: savanna | 15 | 999 | 0.985 | 0.445 | 0.688 |
|  | Grassland: hot (persistently dry) | Grassland: hot (summer drought) | 10 | 999 | 1.915 | 0.034 | 0.366 |
|  | Grassland: hot (persistently dry) | Grassland: warm (persistently dry) | 34 | 999 | 1.774 | 0.058 | 0.366 |
|  | Grassland: hot (persistently dry) | Subtropical: moderately dry winter | 10 | 999 | 1.443 | 0.108 | 0.474 |
|  | Grassland: hot (persistently dry) | Subtropical: no dry season | 12 | 999 | 1.631 | 0.045 | 0.366 |
|  | Grassland: hot (persistently dry) | Temperate: distinctly dry (and hot) summer | 14 | 999 | 1.532 | 0.103 | 0.474 |
|  | Grassland: hot (persistently dry) | Temperate: distinctly dry (and warm) summer | 56 | 999 | 2.626 | 0.001 | 0.044 |
|  | Grassland: hot (persistently dry) | Temperate: no dry season (hot summer) | 11 | 999 | 1.421 | 0.115 | 0.474 |
|  | Grassland: hot (persistently dry) | Temperate: no dry season (mild summer) | 11 | 999 | 1.773 | 0.061 | 0.366 |
|  | Grassland: hot (persistently dry) | Temperate: no dry season (warm summer) | 42 | 999 | 2.617 | 0.004 | 0.066 |
|  | Grassland: hot (persistently dry) | Tropical: savanna | 11 | 999 | 1.055 | 0.336 | 0.599 |
|  | Grassland: hot (summer drought) | Grassland: warm (persistently dry) | 30 | 999 | 0.961 | 0.464 | 0.688 |
|  | Grassland: hot (summer drought) | Subtropical: moderately dry winter | 6 | 999 | 1.012 | 0.510 | 0.701 |
|  | Grassland: hot (summer drought) | Subtropical: no dry season | 8 | 999 | 0.810 | 0.621 | 0.748 |
|  | Grassland: hot (summer drought) | Temperate: distinctly dry (and hot) summer | 10 | 999 | 1.317 | 0.167 | 0.503 |
|  | Grassland: hot (summer drought) | Temperate: distinctly dry (and warm) summer | 52 | 999 | 0.972 | 0.469 | 0.688 |
|  | Grassland: hot (summer drought) | Temperate: no dry season (hot summer) | 7 | 999 | 0.933 | 0.494 | 0.701 |
|  | Grassland: hot (summer drought) | Temperate: no dry season (mild summer) | 7 | 999 | 0.671 | 0.974 | 0.974 |
|  | Grassland: hot (summer drought) | Temperate: no dry season (warm summer) | 38 | 999 | 1.169 | 0.219 | 0.513 |
|  | Grassland: hot (summer drought) | Tropical: savanna | 7 | 999 | 0.867 | 0.715 | 0.823 |
|  | Grassland: warm (persistently dry) | Subtropical: moderately dry winter | 30 | 999 | 1.270 | 0.183 | 0.503 |
|  | Grassland: warm (persistently dry) | Subtropical: no dry season | 32 | 999 | 1.132 | 0.281 | 0.530 |
|  | Grassland: warm (persistently dry) | Temperate: distinctly dry (and hot) summer | 34 | 999 | 1.174 | 0.243 | 0.517 |
|  | Grassland: warm (persistently dry) | Temperate: distinctly dry (and warm) summer | 76 | 999 | 2.732 | 0.002 | 0.044 |
|  | Grassland: warm (persistently dry) | Temperate: no dry season (hot summer) | 31 | 999 | 1.303 | 0.203 | 0.513 |
|  | Grassland: warm (persistently dry) | Temperate: no dry season (mild summer) | 31 | 999 | 0.890 | 0.532 | 0.717 |
|  | Grassland: warm (persistently dry) | Temperate: no dry season (warm summer) | 62 | 999 | 2.953 | 0.002 | 0.044 |
|  | Grassland: warm (persistently dry) | Tropical: savanna | 31 | 999 | 1.067 | 0.352 | 0.609 |
|  | Subtropical: moderately dry winter | Subtropical: no dry season | 8 | 999 | 0.930 | 0.609 | 0.748 |
|  | Subtropical: moderately dry winter | Temperate: distinctly dry (and hot) summer | 10 | 999 | 1.424 | 0.131 | 0.480 |
|  | Subtropical: moderately dry winter | Temperate: distinctly dry (and warm) summer | 52 | 999 | 1.522 | 0.046 | 0.366 |
|  | Subtropical: moderately dry winter | Temperate: no dry season (hot summer) | 7 | 999 | 0.465 | 0.973 | 0.974 |
|  | Subtropical: moderately dry winter | Temperate: no dry season (mild summer) | 7 | 999 | 0.858 | 0.603 | 0.748 |
|  | Subtropical: moderately dry winter | Temperate: no dry season (warm summer) | 38 | 999 | 1.333 | 0.144 | 0.500 |
|  | Subtropical: moderately dry winter | Tropical: savanna | 7 | 999 | 0.753 | 0.946 | 0.974 |
|  | Subtropical: no dry season | Temperate: distinctly dry (and hot) summer | 12 | 999 | 0.937 | 0.460 | 0.688 |
|  | Subtropical: no dry season | Temperate: distinctly dry (and warm) summer | 54 | 999 | 0.884 | 0.615 | 0.748 |
|  | Subtropical: no dry season | Temperate: no dry season (hot summer) | 9 | 999 | 0.720 | 0.933 | 0.974 |
|  | Subtropical: no dry season | Temperate: no dry season (mild summer) | 9 | 999 | 0.775 | 0.805 | 0.901 |
|  | Subtropical: no dry season | Temperate: no dry season (warm summer) | 40 | 999 | 0.979 | 0.430 | 0.688 |
|  | Subtropical: no dry season | Tropical: savanna | 9 | 999 | 0.732 | 0.822 | 0.904 |
|  | Temperate: distinctly dry (and hot) summer | Temperate: distinctly dry (and warm) summer | 56 | 999 | 1.159 | 0.230 | 0.513 |
|  | Temperate: distinctly dry (and hot) summer | Temperate: no dry season (hot summer) | 11 | 999 | 1.287 | 0.203 | 0.513 |
|  | Temperate: distinctly dry (and hot) summer | Temperate: no dry season (mild summer) | 11 | 999 | 0.964 | 0.424 | 0.688 |
|  | Temperate: distinctly dry (and hot) summer | Temperate: no dry season (warm summer) | 42 | 999 | 1.274 | 0.176 | 0.503 |
|  | Temperate: distinctly dry (and hot) summer | Tropical: savanna | 11 | 999 | 1.102 | 0.316 | 0.579 |
|  | Temperate: distinctly dry (and warm) summer | Temperate: no dry season (hot summer) | 53 | 999 | 1.220 | 0.177 | 0.503 |
|  | Temperate: distinctly dry (and warm) summer | Temperate: no dry season (mild summer) | 53 | 999 | 0.747 | 0.852 | 0.922 |
|  | Temperate: distinctly dry (and warm) summer | Temperate: no dry season (warm summer) | 84 | 999 | 1.449 | 0.074 | 0.376 |
|  | Temperate: distinctly dry (and warm) summer | Tropical: savanna | 53 | 999 | 1.150 | 0.224 | 0.513 |
|  | Temperate: no dry season (hot summer) | Temperate: no dry season (mild summer) | 8 | 999 | 0.771 | 0.723 | 0.823 |
|  | Temperate: no dry season (hot summer) | Temperate: no dry season (warm summer) | 39 | 999 | 1.119 | 0.273 | 0.530 |
|  | Temperate: no dry season (hot summer) | Tropical: savanna | 8 | 999 | 0.690 | 0.965 | 0.974 |
|  | Temperate: no dry season (mild summer) | Temperate: no dry season (warm summer) | 39 | 999 | 0.810 | 0.675 | 0.796 |
|  | Temperate: no dry season (mild summer) | Tropical: savanna | 8 | 999 | 0.864 | 0.623 | 0.748 |
|  | Temperate: no dry season (warm summer) | Tropical: savanna | 39 | 999 | 1.145 | 0.233 | 0.513 |
| **Land cover** | Annual crops and highly modified pastures | Built-up | 38 | 999 | 1.212 | 0.180 | 0.569 |
|  | Annual crops and highly modified pastures | Ephemeral and Permanent Water Features | 36 | 999 | 0.716 | 0.909 | 0.979 |
|  | Annual crops and highly modified pastures | Horticultural trees and shrubs | 36 | 999 | 0.671 | 0.954 | 0.989 |
|  | Annual crops and highly modified pastures | Native forests and woodlands | 94 | 999 | 1.383 | 0.084 | 0.482 |
|  | Annual crops and highly modified pastures | Native grasslands and minimally modified pastures | 61 | 999 | 1.908 | 0.011 | 0.308 |
|  | Annual crops and highly modified pastures | Native shrublands and heathlands | 51 | 999 | 1.609 | 0.059 | 0.482 |
|  | Built-up | Ephemeral and Permanent Water Features | 6 | 999 | 0.852 | 0.618 | 0.865 |
|  | Built-up | Horticultural trees and shrubs | 6 | 999 | 1.154 | 0.330 | 0.840 |
|  | Built-up | Native forests and woodlands | 64 | 999 | 0.962 | 0.454 | 0.840 |
|  | Built-up | Native grasslands and minimally modified pastures | 31 | 999 | 1.437 | 0.086 | 0.482 |
|  | Built-up | Native shrublands and heathlands | 21 | 999 | 0.912 | 0.535 | 0.840 |
|  | Ephemeral and Permanent Water Features | Horticultural trees and shrubs | 4 | 999 | 0.723 | 1.000 | 1.000 |
|  | Ephemeral and Permanent Water Features | Native forests and woodlands | 62 | 999 | 0.777 | 0.804 | 0.938 |
|  | Ephemeral and Permanent Water Features | Native grasslands and minimally modified pastures | 29 | 999 | 0.972 | 0.393 | 0.840 |
|  | Ephemeral and Permanent Water Features | Native shrublands and heathlands | 19 | 999 | 0.949 | 0.524 | 0.840 |
|  | Horticultural trees and shrubs | Native forests and woodlands | 62 | 999 | 0.844 | 0.682 | 0.868 |
|  | Horticultural trees and shrubs | Native grasslands and minimally modified pastures | 29 | 999 | 0.845 | 0.679 | 0.868 |
|  | Horticultural trees and shrubs | Native shrublands and heathlands | 19 | 999 | 1.010 | 0.449 | 0.840 |
|  | Native forests and woodlands | Native grasslands and minimally modified pastures | 87 | 999 | 1.832 | 0.025 | 0.350 |
|  | Native forests and woodlands | Native shrublands and heathlands | 77 | 999 | 1.068 | 0.331 | 0.840 |
|  | Native grasslands and minimally modified pastures | Native shrublands and heathlands | 44 | 999 | 1.254 | 0.167 | 0.569 |

**Table S6: Captive vs wild echidna alpha diversity statistics.** PD = Phylogenetic Diversity; ASVs = Amplicon Sequence Variants.

|  | Group 1 | Group 2 | H | p-value | q-value |
| --- | --- | --- | --- | --- | --- |
| **Faith's PD** | captive (n=39) | wild (n=159) | 0.0943 | 0.7587 | 0.7587 |
| **Observed ASV's** | captive (n=39) | wild (n=159) | 0.0019 | 0.9652 | 0.9652 |
| **Shannon's Diversity** | captive (n=39) | wild (n=159) | 0.4516 | 0.5016 | 0.5016 |
| **Pileou's Evenness** | captive (n=39) | wild (n=159) | 1.3932 | 0.2379 | 0.2379 |

**Table S7: Captive vs wild echidna beta diversity statistics.** Green rows = statistically significant.

|  | **Unweighted UniFrac** | | | | | | | |
| --- | --- | --- | --- | --- | --- | --- | --- | --- |
|  | **Group 1** | **Group 2** | **Sample size** | **Permutations** | **pseudo-F** | **p-value** | **q-value** |  |
| **Diet** | Meat | UMD | 29 | 999 | 2.09326 | 0.001 | 0.0017 |  |
|  | Meat | Vetafarm | 30 | 999 | 4.889711 | 0.001 | 0.0017 |  |
|  | Meat | Wombaroo | 28 | 999 | 4.112972 | 0.001 | 0.0017 |  |
|  | Meat | insect | 183 | 999 | 5.190173 | 0.001 | 0.0017 |  |
|  | UMD | Vetafarm | 11 | 999 | 1.612909 | 0.041 | 0.043 |  |
|  | UMD | Wombaroo | 9 | 999 | 1.91877 | 0.043 | 0.043 |  |
|  | UMD | insect | 164 | 999 | 1.983871 | 0.007 | 0.01 |  |
|  | Vetafarm | Wombaroo | 10 | 999 | 2.107347 | 0.031 | 0.0388 |  |
|  | Vetafarm | insect | 165 | 999 | 3.346711 | 0.001 | 0.0017 |  |
|  | Wombaroo | insect | 163 | 999 | 2.87027 | 0.001 | 0.0017 |  |
| **Season** | Autumn | Spring | 104 | 999 | 1.887263 | 0.012 | 0.0144 |  |
|  | Autumn | Summer | 68 | 999 | 1.329154 | 0.118 | 0.118 |  |
|  | Autumn | Winter | 108 | 999 | 2.863068 | 0.001 | 0.003 |  |
|  | Spring | Summer | 90 | 999 | 2.022744 | 0.008 | 0.012 |  |
|  | Spring | Winter | 130 | 999 | 1.941906 | 0.008 | 0.012 |  |
|  | Summer | Winter | 94 | 999 | 3.05829 | 0.001 | 0.003 |  |
| **Zoo** | Perth | Taronga | 39 | 999 | 6.280359 | 0.001 | 0.001 |  |
|  | Perth | wild | 183 | 999 | 5.190173 | 0.001 | 0.001 |  |
|  | Taronga | wild | 174 | 999 | 5.982007 | 0.001 | 0.001 |  |

**Table S8: Captive echidna alpha diversity statistics.** Green rows = statistically significant; PD = Phylogenetic Diversity; ASVs = Amplicon Sequence Variants.

|  | Group 1 | Group 2 | H | p-value | q-value |
| --- | --- | --- | --- | --- | --- |
| **Faith's PD** | Meat (n=26) | UMD (n=5) | 5.5846 | 0.0181 | 0.0362 |
|  | Meat (n=26) | Vetafarm (n=7) | 12.7233 | 0.0004 | 0.0022 |
|  | Meat (n=26) | Wombaroo (n=6) | 7.0513 | 0.0079 | 0.0238 |
|  | UMD (n=5) | Vetafarm (n=7) | 1.4835 | 0.2232 | 0.2679 |
|  | UMD (n=5) | Wombaroo (n=6) | 0.0333 | 0.8551 | 0.8551 |
|  | Vetafarm (n=7) | Wombaroo (n=6) | 1.6531 | 0.1985 | 0.2679 |
| **Observed ASV's** | Meat (n=26) | UMD (n=5) | 6.9302 | 0.0085 | 0.0254 |
|  | Meat (n=26) | Vetafarm (n=7) | 9.1055 | 0.0025 | 0.0153 |
|  | Meat (n=26) | Wombaroo (n=6) | 3.2816 | 0.0701 | 0.1401 |
|  | UMD (n=5) | Vetafarm (n=7) | 0.1660 | 0.6837 | 0.6837 |
|  | UMD (n=5) | Wombaroo (n=6) | 1.6408 | 0.2002 | 0.2403 |
|  | Vetafarm (n=7) | Wombaroo (n=6) | 1.6576 | 0.1979 | 0.2403 |
| **Shannon's Diversity** | Meat (n=26) | UMD (n=5) | 2.1029 | 0.1470 | 0.4411 |
|  | Meat (n=26) | Vetafarm (n=7) | 2.8003 | 0.0942 | 0.4411 |
|  | Meat (n=26) | Wombaroo (n=6) | 1.0280 | 0.3106 | 0.6213 |
|  | UMD (n=5) | Vetafarm (n=7) | 0.0066 | 0.9353 | 0.9353 |
|  | UMD (n=5) | Wombaroo (n=6) | 0.3000 | 0.5839 | 0.8019 |
|  | Vetafarm (n=7) | Wombaroo (n=6) | 0.1837 | 0.6682 | 0.8019 |
| **Pileou's Evenness** | Meat (n=26) | UMD (n=5) | 1.5260 | 0.2167 | 0.6502 |
|  | Meat (n=26) | Vetafarm (n=7) | 1.8636 | 0.1722 | 0.6502 |
|  | Meat (n=26) | Wombaroo (n=6) | 0.6737 | 0.4118 | 0.8236 |
|  | UMD (n=5) | Vetafarm (n=7) | 0.0593 | 0.8075 | 0.8551 |
|  | UMD (n=5) | Wombaroo (n=6) | 0.0333 | 0.8551 | 0.8551 |
|  | Vetafarm (n=7) | Wombaroo (n=6) | 0.0816 | 0.7751 | 0.8551 |

**Table S9: Captive echidna beta diversity statistics.** Green rows = statistically significant.

|  | **Unweighted UniFrac** | | | | | | |
| --- | --- | --- | --- | --- | --- | --- | --- |
|  | **Group 1** | **Group 2** | **Sample size** | **Permutations** | **pseudo-F** | **p-value** | **q-value** |
| **Diet** | Meat | UMD | 31 | 999 | 2.004 | 0.005 | 0.010 |
|  | Meat | Vetafarm | 33 | 999 | 5.085 | 0.001 | 0.003 |
|  | Meat | Wombaroo | 32 | 999 | 3.938 | 0.001 | 0.003 |
|  | UMD | Vetafarm | 12 | 999 | 1.304 | 0.141 | 0.156 |
|  | UMD | Wombaroo | 11 | 999 | 1.409 | 0.156 | 0.156 |
|  | Vetafarm | Wombaroo | 13 | 999 | 1.558 | 0.072 | 0.108 |
| **Season** | Autumn | Spring | 16 | 999 | 1.932 | 0.034 | 0.041 |
|  | Autumn | Summer | 16 | 999 | 1.040 | 0.367 | 0.367 |
|  | Autumn | Winter | 16 | 999 | 2.224 | 0.024 | 0.036 |
|  | Spring | Summer | 28 | 999 | 3.077 | 0.001 | 0.003 |
|  | Spring | Winter | 28 | 999 | 2.345 | 0.001 | 0.003 |
|  | Summer | Winter | 28 | 999 | 3.221 | 0.003 | 0.006 |
| **Sex** | Female | Male | 44 | 999 | 1.345 | 0.120 | 0.120 |
| **Zoo** | Perth | Taronga | 44 | 999 | 6.570 | 0.001 | 0.001 |
| **Breeding season** | No | Yes | 44 | 999 | 2.226 | 0.007 | 0.007 |
